# Functional shifts in immune composition follow lactation stage in human milk

**DOI:** 10.3389/fimmu.2026.1850094

**Published:** 2026-06-24

**Authors:** Jia Ming Low, Melissa Shu Feng Ng, Chen-Shi Lin, Jian-Zhou Cui, Meera K. Shenoy, Sheau Yng Lim, Lu-Yi Ng, Si Min Lang, Wai-Chung Ong, Tamanna Ferdous, Rashi Gupta, Tanusya Murali Murali, Isabelle Tan, Karishma Sachaphibulkij, Yung-Seng Lee, Paul A MacAry

**Affiliations:** 1 Department of Neonatology, Khoo Teck Puat – National University Children’s Medical Institute, National University Hospital, Singapore, Singapore; 2 Department of Paediatrics, Yong Loo Lin School of Medicine, National University of, Singapore, Singapore; 3 Department of Microbiology and Immunology, Yong Loo Lin School of Medicine, National University of Singapore, Singapore, Singapore; 4 Singapore Immunology Network, Agency for Science, Technology and Research, Singapore, Singapore; 5 Immunology Translational Research Program, Yong Loo Lin School of Medicine, National University of Singapore, Singapore, Singapore; 6 Immunology Program, Life Sciences Institute, National University of Singapore, Singapore, Singapore; 7 National University of Singapore (NUS)-Cambridge Immunophenotyping Centre, National University of Singapore, Singapore, Singapore; 8 Antibody Engineering Programme, Life Sciences Institute, National University of Singapore, Singapore, Singapore

**Keywords:** human milk immunology, immune ontogeny, mucosal immunity, single-cell RNA sequencing, transcriptomics

## Abstract

**Introduction:**

Human milk contains diverse immune and non-immune cellular components that change dynamically from early to established lactation. We sought to determine if the milk immune microenvironment exhibits stage-associated changes.

**Methods:**

We performed multi-modal profiling using single-cell RNA sequencing, high-dimensional flow cytometry, and soluble protein assays on a longitudinal cohort of paired maternal peripheral blood samples and milk. Samples were collected across three distinct phases: colostrum, transitional, and mature milk.

**Results:**

Integrated analysis revealed that human milk contains transcriptionally distinct immuno and epithelial cellular populations compared with maternal peripheral blood. Early lactation (colostrum) demonstrated greater neutrophil enrichment of antimicrobial-associated transcriptional programs, including degranulation- and NETosis-associated signatures. Later lactation stages (transition and mature) demonstrated relatively greater effector-memory-associated T-cell transcriptional signatures. Stage-associated differences in soluble immune mediator profiles were also observed, with early recruitment-associated cytokines (CXCL8, CXCL13) declining across lactation while IL-7 increased in mature milk. Cell–cell communication analysis identified inferred lactocyte-associated signalling pathways involving MHC and growth factor-related interactions that differed across lactation stages.

**Conclusion:**

Human milk demonstrates substantial stage-associated remodelling of both cellular and soluble immune components across lactation. Early milk was enriched for neutrophil-associated antimicrobial and inflammatory transcriptional programs, whereas later lactation stages demonstrated stronger effector-memory-associated T-cell signatures together with distinct soluble immune mediator profiles. These findings support the concept that human milk represents a dynamic immune environment that changes across lactation and may expose infants to distinct immune signals during early postnatal life. Further studies will be required to determine the functional significance of these stage-associated immune features and their implications for neonatal health.

## 1 Introduction

Early postnatal life is characterized by heightened susceptibility to infection alongside rapid immune development. Human milk plays an important role in neonatal development by providing nutrients and immunological protection during this critical period (1–3). Primarily, three major stages have been described across lactation – colostrum, transitional milk, and mature milk – reflecting a continuum from early postnatal secretion to a more established phase of milk production, accompanied by temporal changes in milk volume, bioactive factors and nutrients (4). While the transfer of maternal antibodies through milk is well established, far less is known about the cellular components and how they evolve across lactation (5–10). Additionally, most studies have focused on profiling of milk cells at a single, often mature stage of lactation, limiting insight into the longitudinal changes in milk immune composition across early to established lactation (4, 7, 9).

This knowledge gap may be particularly consequential for vulnerable populations such as preterm infants, for whom human milk often represents a major source of immunological support during a period of immune immaturity (1, 11). A central question is whether immune cells present in human milk primarily reflect circulating maternal blood immune populations or exhibit distinct tissue-associated phenotypic features within the lactating mammary environment (2, 10, 12, 13). Immune cells within milk are exposed to a unique mammary tissue environment shaped by local cytokine and chemokine gradients and microbial cues, suggesting that their phenotypes and functions may diverge fundamentally from those of circulating immune cells (6).

Recent studies have applied single-cell RNA sequencing and flow cytometry to characterized individual components of human milk and identified tissue-oriented phenotypes of milk T cells (14–19). Animal models further suggest that maternally derived immune cells in milk can traffic to neonatal lymphoid tissues and influence immune development (14–19). However, longitudinal paired analyses of fresh human milk and matched maternal blood across lactation stages remain limited.

Direct comparison between human milk and paired maternal blood during the early postpartum period may help distinguish milk-associated immune features from systemic maternal immunity and better define how immune cell composition changes across lactation (19). If human milk demonstrates stage-associated differences in cellular and soluble immune composition over time, infants may be exposed to distinct immune environments across lactation stages (20–22).

Recent advances in single-cell RNA sequencing, high-dimensional flow cytometry, and immunoassays now enable high-resolution profiling of immune cells within complex biological systems such as human milk (5, 8, 10, 23, 24). Leveraging these technologies to profile paired maternal milk and blood samples, we sought to determine whether human milk exhibits transcriptionally and immunologically distinct cellular profiles across lactation stage.

## 2 Results

### 2.1 Human milk contains transcriptionally distinct immune and epithelial cellular populations compared with peripheral blood

**Figure 1:**
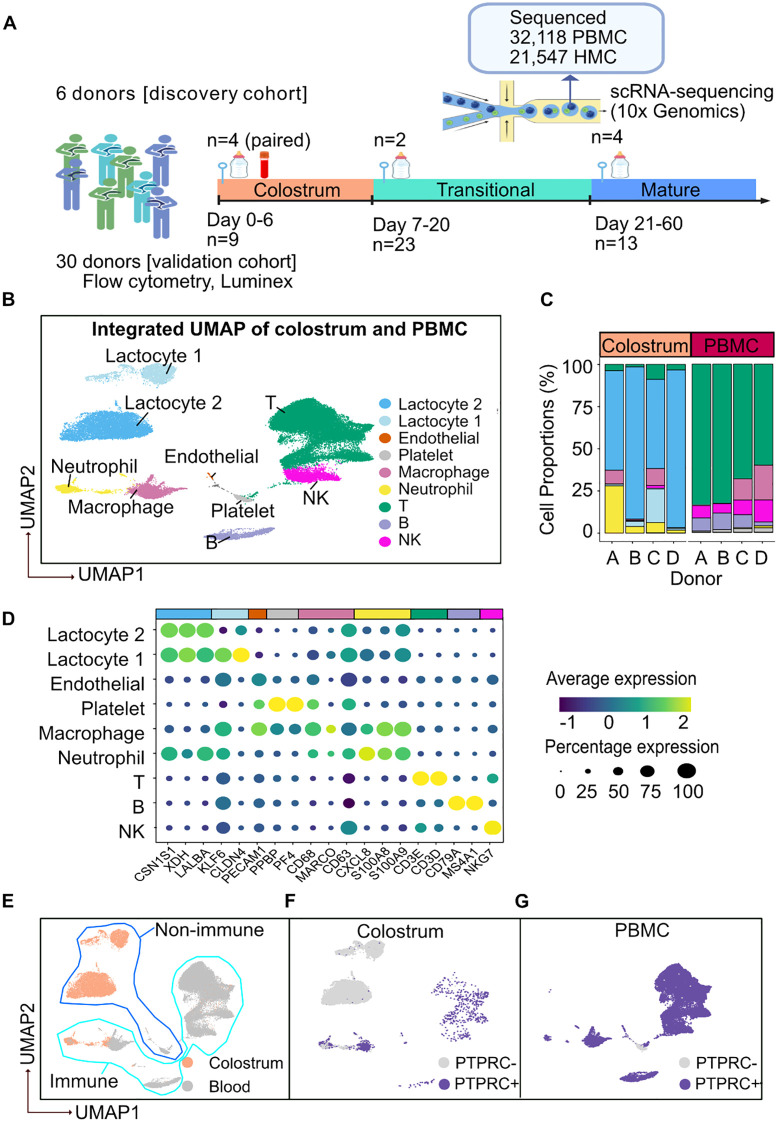
Single-cell landscape of human milk reveals a distinct immune and epithelial cell populations compared with systemic circulation. **(A)** Schematic diagram for the multi-modal experimental setup of cell samples from paired maternal peripheral blood mononuclear cells (PBMC) (n=4) and human milk cells (HMC) (n=10). Longitudinal samples spanned colostrum (n=4), transitional (n=2) and mature (n=4) milk stages. **(B)** Uniform manifold approximation and projection (UMAP) dimensional reduction of colostrum and PBMC from paired donors identifying the constituent immune and epithelial (non-immune) populations. **(C)** Stacked bar plots comparing colostrum and paired PBMC, highlighting the unique cellular composition of the milk compartment. **(D)** Dot plot of canonical marker expression across annotated clusters. Colour represents scaled average expression; size indicates percentage of expressing cells. **(E)** UMAP showing the distinct spatial organization of immune (*PTPRC*) and non-immune populations between colostrum and PBMC. **(F, G)** Feature plots of *PTPRC* expression illustrating the leukocyte fraction within colostrum **(F)** versus PBMC **(G)**. **Figure 1A** was created using BioRender.

To study the dynamic regulation of the milk microenvironment across lactation stages, we established a unique longitudinal cohort of lactating mothers who provided matched samples of blood and milk across different lactation stages (i.e., colostrum, transitional and mature milk) (**Figure 1A**). By processing each freshly expressed milk sample from an entire, single-feed milk expression within a strict six-hour window, we ensured the capture of a high-fidelity snapshot of live cellular activity in milk. Maternal demographic and clinical characteristics, including parity, gestational age at delivery, mode of delivery, gestational diabetes, and maternal infection history, are summarized in **Table 1** and **Supplementary Table 1**. Comprehensive sample accounting across phases and analyses is shown in **Supplementary Tables 2** and **3**, and participant flow is summarized in **Supplementary Figure 1**. No major clinical differences were observed across the n = 30 donors.

**Table 1:** Maternal characteristics of the study cohort (n=30).

Clinical characteristics	n = 30
Maternal age, years (mean, 95% confidence interval (CI))	33.3 (32.7 – 34.0)
Maternal past medical history, if any, n (%)	
Maternal gestational diabetes	5 (16.7)
Maternal pre-eclampsia/pre-existing hypertension	0 (0)
Histologically proven maternal chorioamnionitis	2 (6.6)
First child, n (%)	8 (26.7)
Gender of child, female: male ratio	15:15
Mode of delivery, n (%)	
Normal vaginal delivery	18 (60)
Lower segment Caesarean delivery	12 (40)
Gestational age at delivery, weeks, mean (95% CI)	38 + 3 (37 + 2 – 39 + 3)
Volume of milk provided (median, IQR)	
Colostrum	22 (8.5 – 26)
Transitional milk	38 (28.5–46)
Mature milk	33.5 (22.5–49)

After quality control filtering, a total of 21,547 human milk cells and 32,118 peripheral blood mononuclear cells (PBMC) were retained for downstream analyses. Human milk samples comprised four colostrum donors (Patients A–D), two transitional milk donors (Patients E –F), and four mature milk donors (Patients A–D). Colostrum milk samples yielded between 1764–4535 cells per donor, transitional milk donors yielded 238 and 1983 cells respectively, and mature milk donors yielded between 616 and 3217 cells per donor. Paired maternal peripheral blood mononuclear cell (PBMC) samples from colostrum-stage donors yielded between 6,950 and 9,516 cells per donor following quality control. Overall, milk samples demonstrated greater variability in cellular recovery and transcript complexity across lactation stages compared with blood samples, consistent with the heterogeneous cellular composition of human milk. Median detected genes per cell ranged from 734–1,825 in colostrum milk, 1,332–1,826 in transitional milk, and 952–5,559 in mature milk samples, while median mitochondrial transcript percentages remained low across samples, supporting overall dataset quality. Detailed per-sample QC metrics are provided in **Supplementary Table 4**. The integrated analysis revealed that the presence of both immune (macrophages, neutrophils, B cells, T cells) and non-immune lineages, including two lactocyte subtypes, endothelial cells, and platelets (**Figures 1B, C**). Cell identities were confirmed by canonical marker expression, visualized as a dot plot showing average expression levels and the proportion of expressing cells across major immune and epithelial populations in human milk (**Figure 1D**). Immune cell populations were identified using established markers including T cells (*CD3D, CD3E*), B cell (*CD79A, MS4A1*) and natural killer (NK) cells (*NKG7*). Neutrophils were characterized by high expression of *S100A8/S100A9*, while macrophages expressed canonical markers such as *CD68* and *MARCO*. Non-immune cells were also identified including endothelial cells (*PECAM1*), platelets (*PPBP, PF4*), lactocytes by lactation associated genes (*LALBA, CSN1S1, XDH, KLF6*). These cell identity assignments were supported by concordance with previously published single-cell transcriptomic datasets (10, 24). Stratification by *PTPRC* expression confirmed separation of immune and non-immune populations in milk (**Figure 1E**). UMAP embeddings revealed compartment specific clustering patterns for milk and PBMC datasets across immune and non-immune populations (**Figures 1F, G**). These visualizations were interpreted descriptively and were not used in isolation to infer transcriptional distinctness between compartments. We additionally note that several immune subsets were represented by relatively small paired cell numbers after stratification by compartment and lactation stage, limiting robust subset-level milk-versus-blood differential-expression analyses.

### 2.2 Cellular composition in milk undergoes stage-associated changes as lactation matures

Having established that milk exhibits a unique immunological architecture distinct from circulation, we next investigated whether this microenvironment undergoes programmed evolution as lactation matures. We sought to determine if stage-specific adaptations occur over time as a mother transits from the immediate postpartum period to established breastfeeding.

**Figure 2:**
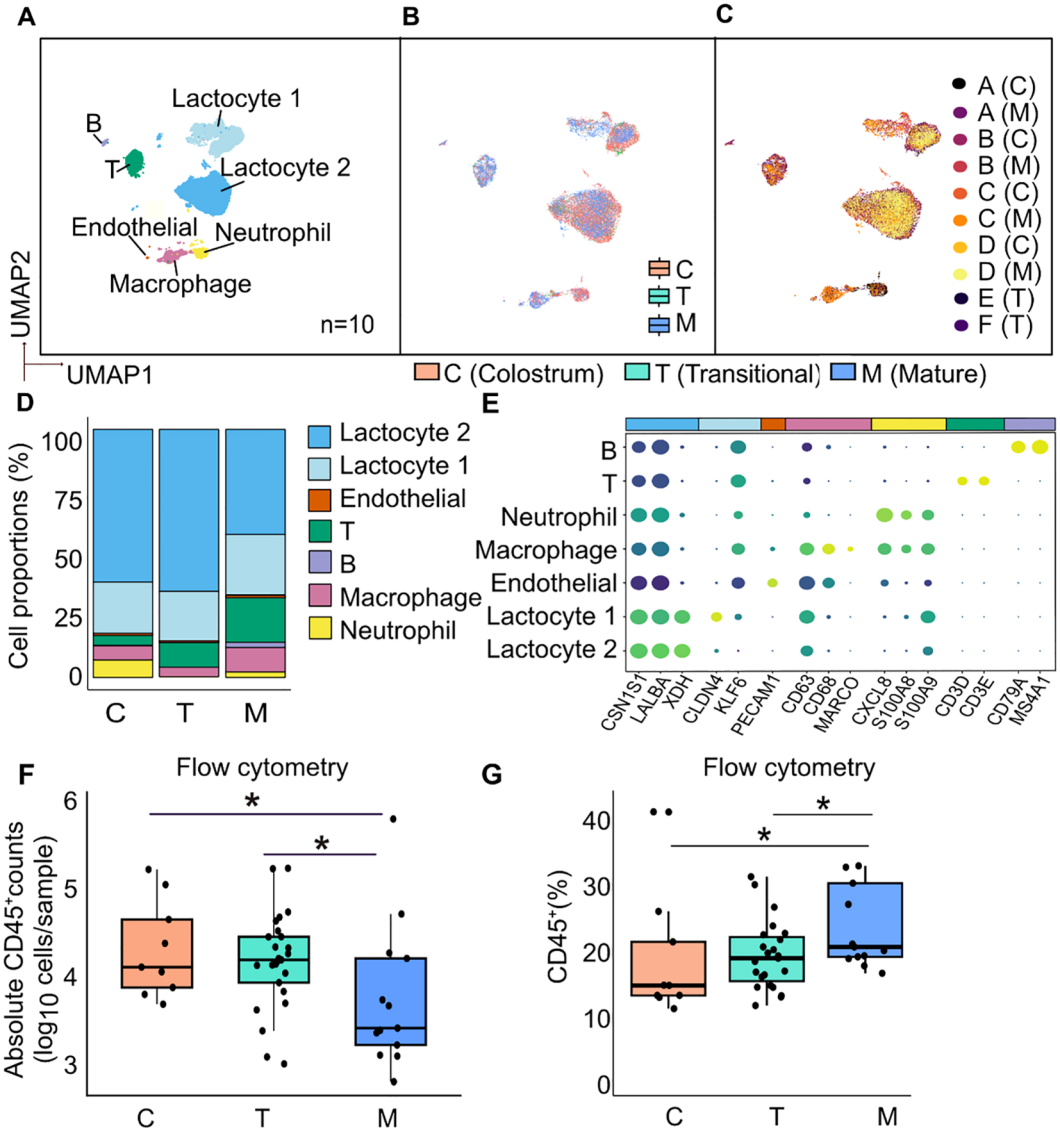
Temporal shifts in human milk immune cell composition across lactation. **(A–C)** Longitudinal integrated UMAP of human milk cells (HMC) across colostrum (C; n=4), transitional (T; n=2), and mature (M; n=4) milk stages. Panels highlight **(A)** annotated cell clusters, **(B)** density distribution by stage, and **(C)** individual sample contributions (n=10). **(D)** Relative proportions of epithelial and immune lineages. **(E)** Dot plot of canonical markers confirming cluster signatures across the lactation continuum. **(F)** Comparison of absolute CD45+ cell count per sample across stages via flow cytometry (colostrum n= 9; transitional n= 23, mature n=13 samples). **(G)** Comparison of CD45+ (immune) cell percentages across stages via (flow cytometry (colostrum n= 9; transitional n= 23, mature n=13 samples). Boxplots show median and interquartile range. Each dot represents one sample. Statistical comparisons across lactation stages were performed using Kruskal–Wallis tests with Benjamini–Hochberg-adjusted Dunn’s multiple-comparisons test. Only statistically significant comparisons are shown (*p < 0.05).

This was done by interrogating the longitudinal shifts in gene expression from 21,547 human milk cells collected across the colostrum, transitional, and mature stages (**Figures 2A, B**). Seven major clusters were identified, including two lactocyte subtypes (Lactocyte 1: high *CLDN4/KLF6*; Lactocyte 2: low/absent *CLDN4/KLF6*) (10, 24), a *PECAM1* endothelial population, and four immune lineages: T cells, B cells, neutrophils, and macrophages/monocytes (**Figure 2A**). After integration and dimensional reduction, cells segregated primarily by type (**Figure 2A**) and breastfeeding stage (**Figure 2B**) rather than donor identity (**Figure 2C**). The stacked bar graphs suggest stage-associated differences in the relative representation of epithelial and immune populations across lactation stages, including relatively greater immune cell representation in mature milk (**Figure 2D**). Clusters were annotated by canonical marker expression to confirm cell (10, 24) identities in **Figure 2E**. Stage-associated transcriptional remodelling was also observed within the myeloid and epithelial compartments of human milk. For mononuclear phagocytes, sub-clustering of the single-cell RNA-sequencing dataset identified two macrophage states alongside a monocyte population (**Supplementary Figure 2B**). In parallel, lactocyte sub-clustering revealed two dominant epithelial subtypes that were conserved across lactation stages (**Supplementary Figure 2C****).**

To corroborate the general cellular shifts observed by single-cell RNA sequencing (**Supplementary Figure 3A**), flow cytometry was performed on 45 additional milk samples across the three lactation stages in the validation phase (**Figure 2F**). The absolute CD45+ immune cell counts per sample decreased significantly across lactation (**Figure 2F**) while the relative proportion of CD45^+^ immune cells increased significantly across lactation (**Figure 2G**). Absolute CD45+ cell counts per sample decreased across lactation, with median concentrations of 18,828 cells/mL (IQR 11,686–57,415) in colostrum, 22,289 cells/mL (13,098–38,304) in transitional milk, and 4,517 cells/mL (3,042–23,079) in mature milk (p<0.05) (**Figure 2F**). In contrast, the relative proportion of CD45+ immune cells rose from a median of 5.6% (IQR 3.5–14.9) in colostrum to 11.4% (IQR 6.5–15.9) in transitional and 13.8% (interquartile range (IQR) 11.7–22.7) in mature milk (p < 0.05) (**Figure 2G**).

The neutrophil-to-lymphocyte ratio (NLR), used here as a descriptive measure of relative neutrophil to lymphocyte representation within human milk, demonstrated a numerical decrease over lactation (**Supplementary Figures 3B, C**) (Flow cytometry data showed colostrum 3.46 → transitional 1.69 → mature 0.44) (p = 0.08). CD3^+^ T cells remained the dominant lymphocyte population (median 43.1%), whereas CD19^+^ B cells were consistently low (median 2.0%) (**Supplementary Figures 2D, E**). T-cell frequencies showed variability across lactation stages (colostrum 34.8% → transitional 35.9% → mature 55.8%; p = 0.78) (**Supplementary Figure 2E**).

### 2.3 Neutrophils transition from an antimicrobial effector program towards a regulatory state over the course of breastfeeding

**Figure 3:**
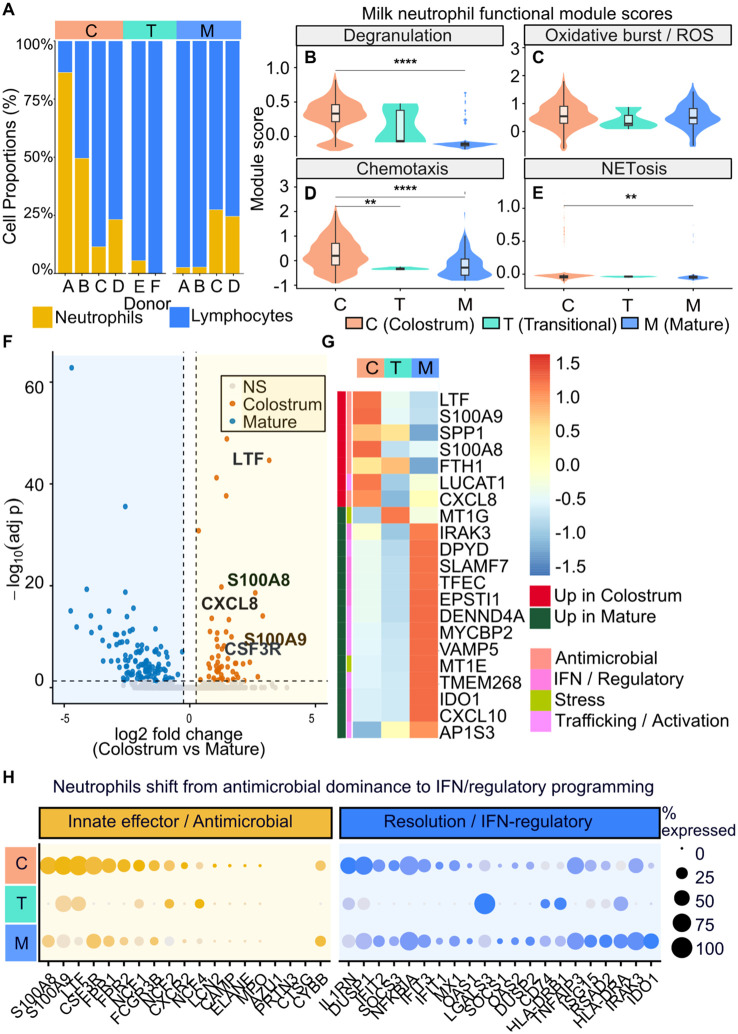
Milk neutrophils undergo a functional switch from antimicrobial effector states to interferon (IFN)-regulatory programming. **(A)** Individual donor plots of neutrophil-to-lymphocyte ratio across colostrum (C; n=4), transitional (T; n=2) and mature (M; n=4) milk stages. **(B–E)** Violin plots representing transcriptomic module scores for **(B)** degranulation, **(C)** oxidative burst, **(D)** chemotaxis, and **(E)** NETosis. Significant downregulation of degranulation, chemotaxis and NETosis functions are observed as lactation progressed. **(F)** Volcano plot comparing colostrum and mature milk neutrophils. Key antimicrobial and granule-associated genes (*LTF*, *S100A8*, *S100A9*, *CXCL8*, and *CSF3R)* are significantly enriched in colostrum. **(G)** Heatmap of top differentially expressed genes categorized by functional pathways demonstrating the transition from antimicrobial dominance to regulatory and interferon-associated signalling. **(H)** Dot plot visualizing the expression of innate effector/antimicrobial markers versus resolution/IFN-regulatory markers across stages. For violin plot comparisons, statistical analyses were performed using Kruskal–Wallis tests with Dunn’s multiple-comparisons tests. Only statistically significant comparisons are shown (**p < 0.01; ****p < 0.0001).

To further characterize the functional properties of early innate immunity in milk, we specifically analysed neutrophil transcriptional programs across lactation. Visual inspection of the paired scRNA-seq dataset suggested inter-individual variability in neutrophil and lymphocyte proportions across lactation (**Figure 3A**). Functional module scoring revealed that colostrum neutrophils were enriched for antimicrobial effector programs, including degranulation, chemotaxis, and NETosis, compared with mature milk neutrophils (degranulation *p* < 0.0001, chemotaxis *p* < 0.0001, NETosis *p* < 0.01) (**Figures 3B, D, E**). These programs were characterized by increased expression of classical antimicrobial and granule-associated genes including *MPO, ELANE, PRTN3, CTSG, AZU1, LTF, CXCR2, FPR1/2, FCGR3B, and CSF3R*. In contrast, neutrophils in mature milk displayed reduced expression of these antimicrobial signatures and relatively greater representation of oxidative burst–associated transcripts, including *NCF1, NCF2, NCF4, CYBB*, and *SOD2* (**Figure 3C**).

Differential expression analysis comparing colostrum and mature milk further supported this stage-associated shift. Colostrum neutrophils were enriched for classical antimicrobial effector genes including *LTF, CXCL8, CSF3R, S100A8, S100A9* (**Figure 3F**). Assessment of the top 10 DEGs demonstrated stage-associated changes in neutrophil transcriptional programs across lactation with mature milk neutrophils showing relatively greater expression of interferon- and immune-response associated transcripts (**Figure 3G**). Direct visualization of antimicrobial effector versus interferon/regulatory-associated genes further illustrated this pattern: colostrum neutrophils preferentially expressed antimicrobial genes, whereas mature milk neutrophils showed increased expression of transcripts including *CXCL10, IDO1, IRAK3*, and *SLAMF7* (**Figure 3H**). As these genes may also be induced by inflammatory, interferon-mediated, or tissue stress responses, these findings should not be interpreted as definitive evidence of a regulatory neutrophil state.

Gene Ontology (GO) enrichment analysis substantiated this transition (**Supplementary Figures 4A, B**). Genes upregulated in colostrum were enriched for pathways related to chemotaxis, leukocyte migration, and neutrophil recruitment, whereas genes enriched in mature milk were associated with regulation of innate immunity, pattern-recognition receptor signalling, and immune activation pathways.

These findings demonstrate stage-associated differences in milk neutrophils transcriptional signatures across lactation with colostrum enriched for antimicrobial-associated gene programs and later lactation stages demonstrating relatively greater interferon and immune-response associated transcriptional signatures. However, the current analyses do not distinguish whether these signatures arise from transcriptional remodelling within shared neutrophil populations or differential representation of distinct neutrophil subsets across lactation stages.

### 2.4 Milk is enriched for *CD8+* T cells and shows progressive acquisition of effector and memory T cell programs

**Figure 4:**
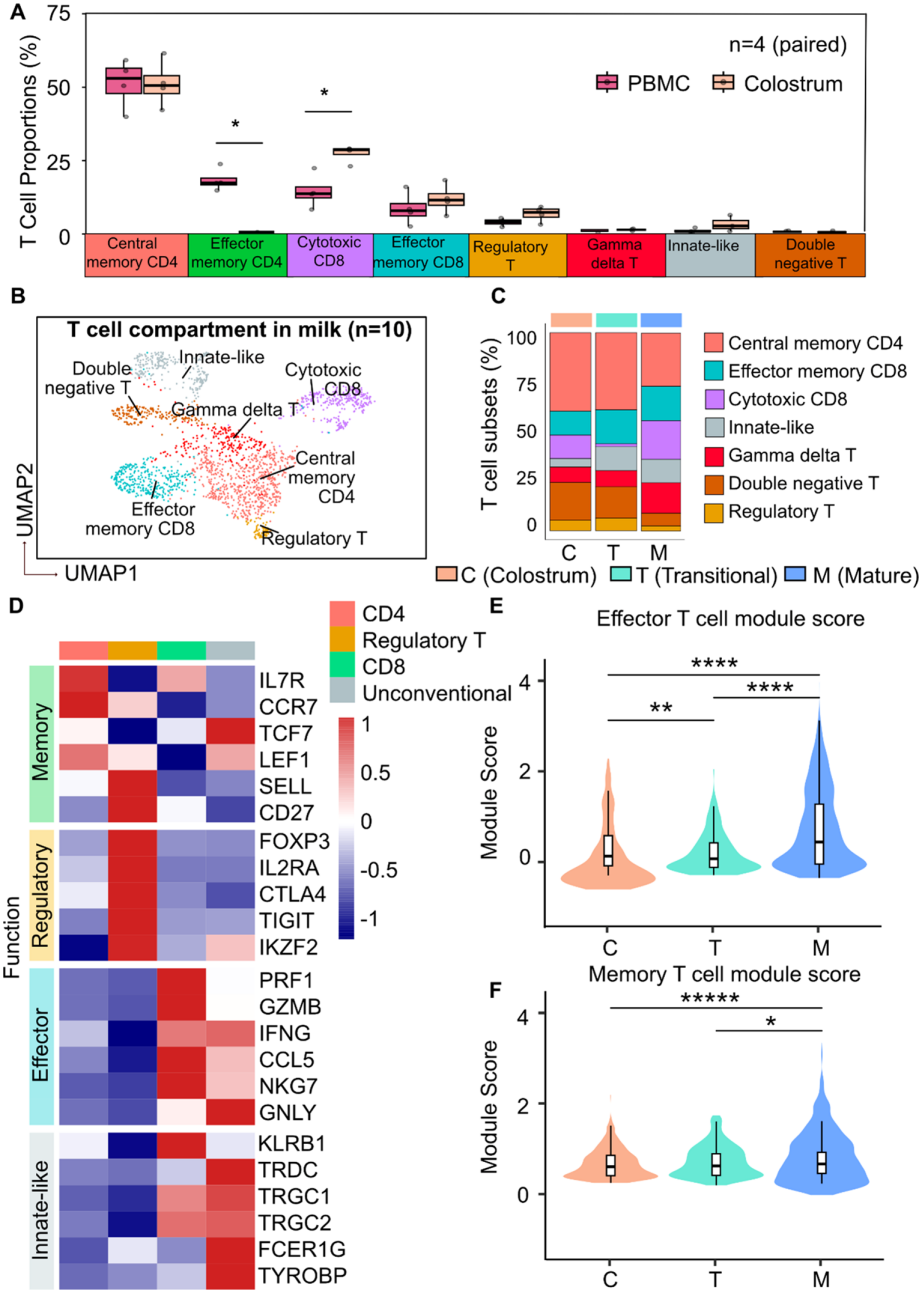
Milk is enriched for cytotoxic CD8+ T cell subsets and exhibits increasing effector and memory T cell functional signatures across lactation. **(A)** Comparison of T cell subset proportions between paired PBMC and colostrum (n=4 pairs) where milk T cells exhibit enrichment of cytotoxic CD8+ relative to PBMC. **(B)** UMAP visualization of integrated single-cell RNA sequencing data from human milk T cells (n = 10 donors), showing distinct clustering of major T-cell subsets. **(C)** Distribution of T cell subsets across lactation stages. **(D)** Heatmap of subset-level gene expression showing canonical functional programs across major T-cell compartments in milk. Values represent row-wise Z-scored average expression. **(E)** Increase in effector T cell module score across lactation. **(F)** Increase in memory T cell module score across lactation. Statistical analyses were performed using Kruskal–Wallis tests with Dunn’s multiple-comparisons tests. Only statistically significant comparisons are shown. Only statistically significant comparisons are shown (*p<0.05; **p < 0.01; ****p < 0.0001; *****p<0.00001).

The transition observed within the neutrophil compartment led us to question whether the adaptive arm of the milk niche undergoes a similar functional maturation. We next investigated T cell composition and functional states within the milk compartment compared to PBMC. In contrast to paired PBMC, human milk is enriched for CD8+ subsets, specifically cytotoxic CD8+ T cells (**Figure 4A**). This divergence indicates a highly selective compartment where the lactating breast preferentially recruits and retains specific T cells subsets rather than simply reflecting systemic T cell composition.

While there is a contraction in neutrophil-to-lymphocyte ratios as milk matures, the internal distribution of T cell subsets remains relatively stable from colostrum to mature milk. Sub-clustering of the milk T cells in the single-cell RNA-sequencing dataset identified diverse T cell states, including central memory *CD4+* T cells, effector and cytotoxic *CD8+* T cells, innate-like, γδ T cells, double-negative T cells, and regulatory T cells (**Figures 4B, C**). Longitudinal flow cytometry confirmed this proportion stability, with no significant shifts in CD4+ or CD8+ frequencies (*CD4+* frequencies ranged from 52.6–70.1%, while *CD8+* frequencies ranged from 3.9–14.7%; p = 0.45) (**Supplementary Figure 2D**). This consistency suggests that the lactating breast maintains a relatively stable T cell composition throughout the duration of lactation.

To further define T cell populations within this stable niche, we analysed the expression of canonical lineage markers across these subsets (**Figure 4D**). This transcriptomic profiling demonstrated the presence of specialized functional states: memory CD4+ T cells were defined by high expression of *IL7R* and *CCR7*, while the regulatory T cell (Treg) cluster exhibited a stringent signature of *FOXP3*, *IL2RA*, and *CTLA4*. Similarly, the CD8+ population displayed a robust cytotoxic profile characterized by *GZMB*, *PRF1*, and *IFNG*, and the innate-like population was validated by the expression of unconventional T-cell markers *TRDC* and *TRGC1/2*. This specialized T-cell identities persist throughout the longitudinal remodelling of the broader milk microenvironment. We then performed module scoring to evaluate if these T cells undergo functional remodelling over time. Across lactation, we observed a progressive increase in both effector (*GZMB, PRF1, IFNG, GNLY, NKG7, CCL5*) and memory (*IL7R, CCR7, TCF7, LEF1*, *SELL*) T cell module scores from colostrum to mature milk (p < 0.0001 and p<0.00001, respectively) (**Figures 4E, F**). This suggests that as lactation matures, the milk T cell niche is not merely maintained but also enhanced for immediate effector response.

### 2.5 Soluble immune milieu of milk shift from inflammatory recruitment towards T cell maintenance and trafficking

We reasoned that the longitudinal enhancement of T cell effector and memory programs is associated with stage-specific soluble immune profiles which refine the mucosal niche as lactation progresses. This functional redirection is not isolated to the adaptive arm; rather, it reflects a global transitional in the milk microenvironment where even the innate compartment, specifically neutrophils, adopts a progressively regulatory and immunomodulatory phenotype.

**Figure 5:**
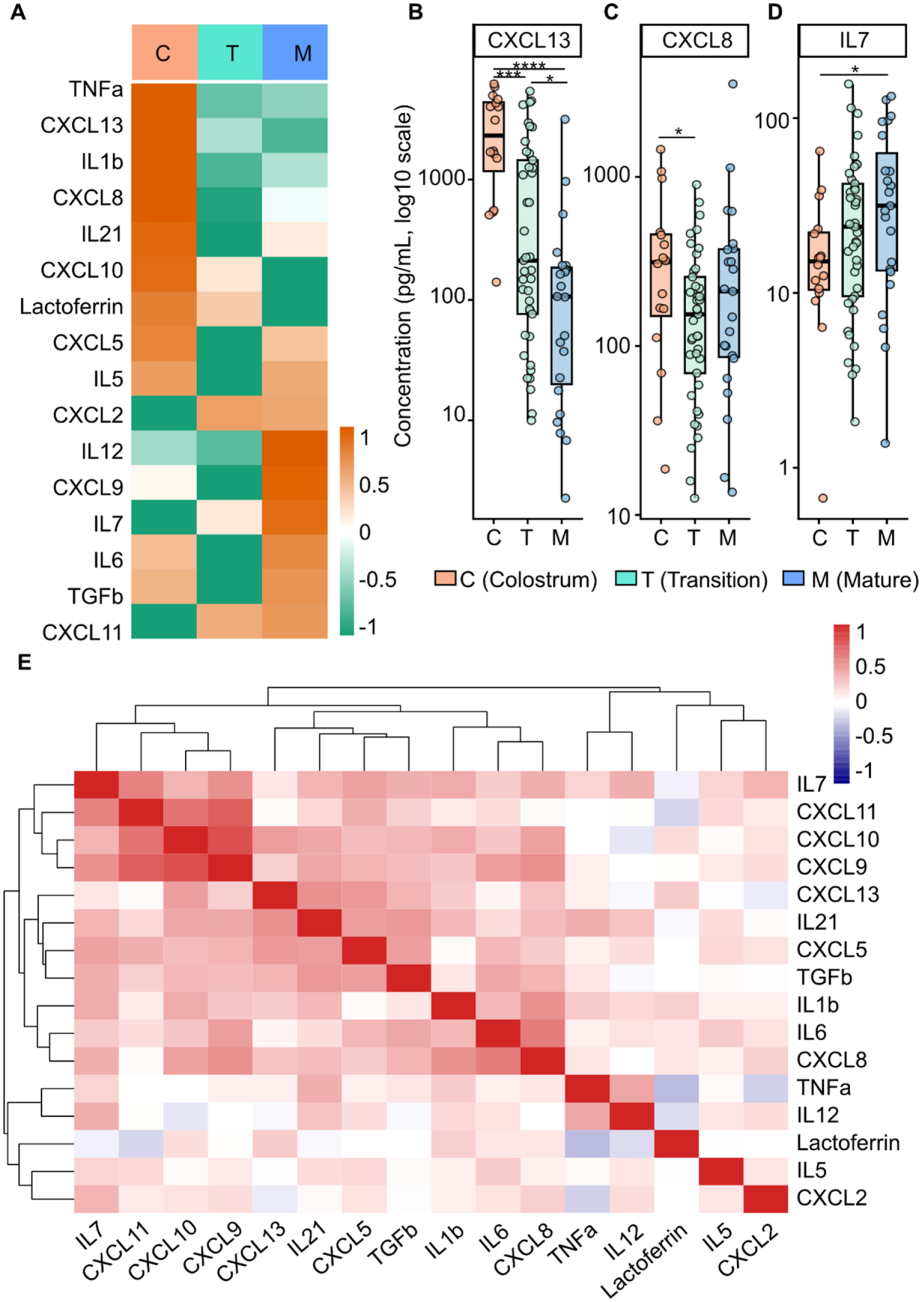
Temporal transition of milk cytokines and chemokines from recruitment to homeostatic maintenance. **(A)** Heatmap of z-score–normalized cytokine and chemokine abundance demonstrating a shift from pro-inflammatory recruitment to homeostatic signalling. **(B–D)** Concentrations of key signalling factors CXCL13 **(B)**, CXCL8 **(C)**, and IL-7. **(D)** across timepoints (n=30). Note the significant decline in recruitment factors (CXCL13/8) contrasted by the significant longitudinal increase in the T cell survival factor IL-7. (pg/mL, log_10_ scale). **(E)** Hierarchical clustering of the cytokine correlation matrix reveals coordinated expression of IL-7 with CXCR3 ligands (CXCL9–11) suggests a microenvironment that supports T-cell survival while actively recruiting effector T cells. Statistical comparisons across lactation stages were performed using Kruskal–Wallis tests with Benjamini–Hochberg-adjusted Dunn’s multiple-comparisons test. Only statistically significant comparisons are shown (*p<0.05; ***p < 0.001; ****p < 0.0001).

We profiled the soluble immune milieu of human milk across lactation and identified stage-associated differences in cytokine and chemokine composition (**Figure 5**). Colostrum was enriched for inflammatory and recruitment-associated mediators including *TNFα, CXCL13, IL1β, CXCL8, IL21*, and *CXCL10*. Transitional milk showed a mixed profile with partial reduction of early inflammatory mediators and emergence of select chemokines reflecting immune rebalancing such as *CXCL10, CXCL 11* and *IL7*. In contrast, mature milk demonstrated relatively higher concentrations of *IL12, CXCL9, IL7, IL6, TGFβ*, and *CXCL11*. These findings indicate substantial temporal remodelling of the soluble immune environment across lactation (**Figure 5A**).

To quantitatively map the evolution of the milk microenvironment, we analysed the longitudinal expression of the dominant cytokines, revealing a divergence between early and mature milk signatures. While recruitment-associated factors, including the B cell and T cell chemoattractant *CXCL13* and the primary neutrophil recruiter *CXCL8*, declined significantly as lactation matured (p < 0.0001; **Figures 5B, C**), we observed a significant, stage-specific increase in the T cell survival factor *IL-7* (p < 0.05; **Figure 5D**).

This reciprocal relationship suggests a programmed functional switch in the mammary secretome: an initial, high-intensity recruitment phase is succeeded by a specialized maintenance phase. By upregulating homeostatic signals like *IL-7*, the milk environment may support maintenance of the T cell niche, even as the early inflammatory surge of lactogenesis subsides. Hierarchical clustering further revealed that *IL-7* co-segregates with a lymphoid-maintenance module (*CXCL9, CXCL10, CXCL11*), suggesting a coordinated shift toward a homeostatic niche (**Figure 5E**).

To contextualize these protein-level changes, we examined the transcriptional activity of the corresponding ligands and receptors across milk cell populations (**Supplementary Figures 5A, B**). This analysis revealed a high degree of concordance between the soluble secretome and cellular gene expression. In early milk, consistent with an observed surge in *CXCL8*, transcripts for this chemokine were localized primarily within the neutrophil and macrophage populations (**Supplementary Figure 5A**). Simultaneously, higher expression of its cognate receptors, *CXCR1* and *CXCR2* in neutrophils suggest an autocrine feedback loop driving early innate recruitment. As lactation progressed towards the mature stage, we observed a transcriptional shift towards maintenance and regulation. Notably, lactocyte 1 and lactocyte 2 emerged as key sources of *LTF* (Lactoferrin) and *TGFB1*, while *IL7* transcripts were notably present in B cells and lactocytes during transitional and mature phases.

While ligands like *CXCL13* declined, the adaptive arm showed sustained or increased expression of receptors for homeostatic cues. T cells maintained high levels of *IL7R* and *IL2RG* across all stages, ensuring they remained primed to receive the survival signals that increase in mature milk (**Supplementary Figure 5B**). Finally, the broad expression of *TGFBR1* and *TGFBR2* across nearly all immune and epithelial compartments in mature milk indicates a systemic shift toward a regulatory environment, aligning with the homeostatic niche identified in our hierarchical clustering (**Supplementary Figure 5B**). By aligning the soluble secretome with these cellular transcriptional programs, we demonstrate that the mammary gland shows changes in both the signals and receivers to sustain immune homeostasis.

Exploratory ligand–receptor inference analysis identified potential stage-associated differences in predicted epithelial–immune interaction networks across lactation. As these analyses are computationally inferred and were not experimentally validated, they are presented as hypothesis-generating observations in the **Supplementary Materials**. Global network analysis identified Lactocyte 2 as primary signalling hub with the highest predicted outgoing signalling strength toward immune populations, particularly T cells (**Supplementary Figures 6A-C**, **7A–D**). Pathway-level inference suggested that Lactocyte 2 may contribute to signalling programs involving *MHC-I, MHC-II, SPP1, GRN*, and *VISFATIN* (**Supplementary Figure 7E**). Stage-resolved analyses revealed dynamic restructuring of these communication networks across lactation. Early lactation was characterized by relatively stronger neutrophil-associated signalling, whereas there was progressively stronger inferred Lactocyte 2 to T cell interactions in mature milk (**Supplementary Figure 6D**).

In addition, gene ontology enrichment analysis identified pathways related to leukocyte chemotaxis, oxidative metabolism, nucleotide biosynthesis, lipid localization, and positive regulation of inflammatory responses as enriched in mature milk compared with colostrum (**Supplementary Figure 7F**). These transcriptional shifts coincided with increasing inferred epithelial–immune ligand–receptor interactions and rising *IL-7* concentrations, suggesting that the mammary epithelial signalling environment across lactation were associated with increased T-cell effector and memory-associated transcriptional signatures.

Collectively, these findings demonstrate that the cellular immune landscape of human milk undergoes temporal remodelling across lactation, transitioning from an early neutrophil-dominated antimicrobial environment toward a later epithelial-associated signalling milieu linked to adaptive T-cell activation.

## 3 Discussion

The availability of fresh colostrum samples paired with contemporaneous maternal blood provided a rare opportunity to examine the immune landscape of early human milk using an integrated multi-modal platform. This unique sampling window allowed us to revisit a well-recognized biological process—maternal immune transfer through milk—through a new lens, capturing cellular and molecular programs that are rarely accessible during the immediate postpartum period.

Historically, human milk research has largely examined nutritional composition and immunological components as separate domains (5, 8, 10, 25). Our findings challenge the traditional view of human milk as either a static nutritional product or a passive filtrate of maternal blood. Instead, we demonstrate that human milk represents a transcriptionally distinct immuno-epithelial immune compartment. Beyond confirming tissue-adapted T-cell features reported in prior studies, our analyses reveal that human milk functions as a dynamic environment that demonstrates temporally distinct immune features across lactation stages.

Specifically, we observed stage-associated differences in the cellular composition and transcriptional profiles of human milk across lactation. Early milk demonstrated greater representation of neutrophils enriched for antimicrobial and migratory transcriptional programs, whereas later lactation stages showed relatively greater lymphocyte representation and effector-memory-associated T-cell signatures. Mature milk neutrophils additionally exhibited increased expression of interferon- and immune-response-associated transcripts. Within the T-cell compartment, colostrum contained diverse tissue-adapted subsets—including regulatory, innate-like, and γδ T cells. These cellular changes were accompanied by stage-associated differences in soluble immune mediators, reflecting dynamic changes in both cellular and soluble components of human milk across lactation.

Our findings are consistent with recent studies demonstrating that human milk T cells exhibit tissue-associated and effector memory phenotypes distinct from circulating maternal blood T cells. In particular, the enrichment of cytotoxic and regulatory T cell programs observed in our dataset aligns with prior single cell analyses of human milk T cells reported by Saager et al. (22). While several major immune and epithelial populations were identified, we did not detect fibroblasts and plasma cells clusters within our dataset, which may reflect their relatively low abundance in expressed human milk, preferential localization within the mammary tissue or under representation during cell isolation and quality-control filtering. Future studies with deeper sequencing and larger cohorts may better resolve these rare populations.

The importance of mother’s own milk (MOM), particularly for preterm infants who experience profound immune immaturity and face increased risk of necrotizing enterocolitis (NEC) and late-onset sepsis remains well established. Our findings suggest that fresh MOM contains dynamically changing cellular and soluble immune components across lactation stages. Whether preservation of these stage-associated immune features influences clinical outcomes in high-risk infants remains an important question for future investigation. When MOM is unavailable, our findings additionally raise questions regarding how donor milk processing methods may influence the cellular and soluble immune composition of milk. Current milk banking practices rely predominantly on Holder pasteurization (27), which, while ensuring microbiological safety, has been reported to reduce viable immune cells and alter certain heat-labile bioactive components. Alternative processing approaches such as high-pressure processing may better preserve selected immune and bioactive factors (28), although the biological and clinical significance of preserving these components remains incompletely understood. Further studies will be required to determine whether preservation of stage-associated immune features or lactation-stage matching meaningfully influences neonatal outcomes.

Several limitations of this study warrant consideration. Ethical constraints precluded direct assessment of the fate or function of milk-derived immune cells in infants. Flow cytometry analyses focused on broad lineage markers due to limited cell numbers, limiting detailed characterization of rare immune subsets. In addition, our cohort consisted of lactating mothers in Singapore, which may limit generalizability to other populations. Maternal clinical characteristics including gestational diabetes and peripartum infections were not specifically controlled for and may contribute to variability in milk immune composition. The cohort was also not powered to assess parity-specific effects on milk immune composition, although recent work by Virassamy et al. suggests that prior pregnancy and lactation may influence mammary T-cell populations and contribute to inter-individual variability in human milk immune profiles (26).

Participants were instructed to completely express a single breast during collection to obtain a representative full-feed milk sample; however, milk expression volumes also varied across donors and lactation stages. As analyses were performed on recovered viable cells for each human milk sample rather than normalized absolute cell counts per mL of milk, differences in recovered cell numbers may reflect variation in milk volume, biological cellularity, and/or technical recovery efficiency. Subclinical mastitis was not formally assessed and therefore cannot be excluded fully as a contributor to some observed interferon-associated transcriptional signatures.

Given the modest donor numbers within the discovery scRNA-seq cohort, stage associated transcriptomic finding should be interpreted as exploratory and hypothesis-generating, particularly where not independently corroborated by flow cytometry or soluble mediator analyses. Milk versus blood comparisons were performed primarily to provide transcriptional context rather than definitive quantitative compartmental analyses. One limitation of this study is that several immune subsets outside the T cell compartment were represented by relatively small cell numbers after stratification by compartment and lactation stage. This limits the robustness of subset-level differential gene expression analyses between blood and colostrum. As such, transcriptional distinctness could not be conclusively established for all immune populations and will require validation in larger cohorts with deeper immune cell sampling. In addition, differential expression analyses were performed at the transcriptomic level without donor-level mixed effects modelling. Finally, the observed neutrophil transcriptional changes across lactation represent stage-associated differences in gene expression profiles, and future functional studies will be required to determine whether these signatures correspond to altered antimicrobial or immunomodulatory activity.

In summary, our data demonstrate substantial stage-associated differences in the cellular and soluble immune composition of human milk across lactation. Early milk was enriched for neutrophil-associated antimicrobial and inflammatory transcriptional programs, whereas later lactation stages demonstrated relatively greater lymphocyte representation and effector-memory-associated T-cell signatures. Together, these findings support the concept that human milk represents a dynamic immune environment that changes across lactation. Further studies will be required to determine the functional significance of these stage-associated immune features and their potential implications for neonatal health and nutrition.

## 4 Materials and methods

### 4.1 Study cohort

This prospective cohort study enrolled lactating mothers from November 2023 to November 2024 at the National University Hospital, Singapore. The study involved two phases, discovery phase and the validation phase. The discovery phase comprised part of the study cohort (n=6). The samples included serial milk samples collected across three lactation stages (colostrum (day 0 – 6), transitional (day 7 – 20), and mature (day 21 – 60) stages), and the maternal blood sample paired with colostrum (available from n=4), for integrated immune profiling by single-cell RNA sequencing (10x Genomics). Because donors contributed multiple lactation-stage samples, the discovery phase comprised 10 milk samples derived from 6 donors. Subsequently the validation phase comprised the whole study cohort (n=30). The samples included serial milk samples collected across three lactation stages for analysis by flow cytometry and multiplex cytokine (Luminex) profiling to validate cellular and soluble immune features across stages (**Figure 1A**). Overall, the discovery phase generated 10 milk samples for scRNA-sequencing, while the validation phase generated 45 milk samples from 30 donors (**Supplementary Tables 2**, **3**). Inclusion criteria were maternal age >21 years, intention to breastfeed, delivery of a term infant, absence of regular medication use, and no known primary immune deficiency or chronic illness. A CONSORT-style flow diagram summarizing participant enrolment, the two phases, and sample distribution across analyses is provided in **Supplementary Figure 1**. Detailed accounting of individual donors, sample availability across lactation stages, and inclusion in each analytical modality is provided in **Supplementary Tables 2** and **3**.

### 4.2 Sample collection: human milk and blood

Participants were instructed to fully empty one breast before a scheduled feed to obtain representative milk samples. Trained staff provided guidance on hygienic collection, including handwashing and use of sterile containers. To minimize technical and sampling variability across lactation stages, milk collection was standardized across all participants. Mothers were instructed to completely empty a single breast during collection to obtain a representative composite milk sample rather than isolate foremilk or hindmilk fractions. Up to 50 mL of fresh milk was collected by breast pump or manual expression and processed within six hours of collection. Samples across all lactation stages were not available for all participants due to logistical and maternal factors. For the discovery phase, paired maternal blood samples were additionally collected at the colostrum stage for integrated immune profiling. Clinical metadata collected included maternal age, parity, gestational age at delivery, mode of delivery and lactation stage. Mothers with clinically apparent mastitis, active breast inflammation, febrile illness at time of sampling were excluded. No participants reported symptoms suggestive of clinical mastitis during collection.

### 4.3 Sample processing: human milk and blood

#### 4.3.1 Isolation of plasma and PBMC

Peripheral blood (1.5–4 mL) was collected in EDTA tubes and diluted with PBS to a final volume of 4 mL. Samples were layered over Ficoll (Cytiva, CAT#GE17-1440-02) and centrifuged to isolate peripheral blood mononuclear cells (PBMC). Plasma was aspirated and stored at –80 °C. The buffy coat was washed with PBS, followed by red blood cell lysis using ACK buffer (Gibco, CAT#A1049201). After additional washing, the PBMC pellet was split into 2 fractions, one fraction was resuspended in freezing medium (10% DMSO in FBS), aliquoted at 1×10^6^ cells per vial, cryopreserved overnight at –80 °C, and subsequently transferred to liquid nitrogen for long-term storage. The other aliquot was processed within six hours of collection to preserve cell viability and minimize ex vivo transcriptional artefacts. Single cell suspensions were prepared immediately following isolation and loaded onto the 10x Genomics Chromium 3′ v3.1 platform.

#### 4.3.2 Human milk cell isolation

Human milk samples were centrifuged at 400 × *g* for 10 minutes at 22 °C to separate cells from the fat and aqueous layers. The cell pellets were washed by removing the fat and supernatant and resuspending in 10 mL of phosphate buffered solution (PBS) before transferring to a new 15 mL tube and centrifuging at 400 × *g* for 10 minutes at 22 °C thrice. Fresh samples were processed within six hours of collection to preserve cell viability and minimize ex vivo transcriptional artefacts. Single cell suspensions were prepared immediately following isolation and loaded onto the 10x Genomics Chromium 3′ v3.1 platform.

### 4.4 Sample analysis: discovery phase and validation phase

#### 4.4.1 Single-Cell RNA sequencing

In the discovery phase, single-cell RNA sequencing of fresh human milk samples (n=10) and paired PBMC samples (n=4) was performed using the 10x Genomics Chromium 3′ v3.1 platform according to the manufacturer’s protocol (10x Genomics, Pleasanton, CA, USA). Cell suspensions were loaded onto the Chromium Controller with a target capture of 5,000 cells per sample. Single-cell gene expression libraries were prepared according to the manufacturer’s protocol and sequenced on an Illumina NovaSeq X Plus platform using 150 bp paired-end reads. Libraries were sequenced on an Illumina NovaSeq X Plus system and processed with Cell Ranger v7.1.0 against the GRCh38 human reference genome. FASTQ generation, alignment, barcode assignment, and unique molecular identifier (UMI) counting were performed using default Cell Ranger parameters. Initial count matrices were imported into Seurat v4.4.0 for downstream analysis in R v4.3.1. Quality control filtering was performed independently for each sample prior to integration. Cells with >10% mitochondrial reads, <200 detected genes, or <500 unique molecular identifiers were excluded, yielding 21,547 milk cells and 32,118 PBMCs for downstream analysis. Detailed per sample quality control metrics, including total cells retained, median genes per cell, median UMIs per cell and mitochondrial transcript percentages are summarized in **Supplementary Table 4**.

Following initial quality-control filtering, ambient RNA contamination was assessed and corrected using SoupX v1.6.2 given the high ambient RNA background associated with human milk samples. Estimated contamination fractions were subtracted from count matrices prior to normalization. Computational doublet detection was subsequently performed on each sample using DoubletFinder v2.0.3, and predicted doublets were removed before downstream analyses.

Milk and PBMC samples were integrated within a single Seurat object to enable direct cross-compartment comparisons, rather than analysed as separate integrated objects. To minimize overcorrection of biologically distinct populations, preliminary annotations of major cell types were first identified within each compartment separately, and concordance of annotations before and after integration was manually reviewed and confirmed. Data normalization and variance stabilisation were performed using SCTransform in Seurat v4.4.0. Integration features were selected using Seurat’s SelectIntegrationFeatures function (nfeatures = 2000) followed by anchor identification using FindIntegrationAnchors and dataset integration with IntegrateData according to the Seurat SCTransform integration workflow. Principal component analysis (PCA) was subsequently performed on the integrated dataset, after which Harmony v1.2.0 was applied as an additional batch-correction step to reduce donor-specific variation prior to downstream clustering and UMAP visualization. Shared nearest-neighbour graphs were constructed using FindNeighbors, and graph-based clustering was performed using FindClusters with a clustering resolution of 0.6 unless otherwise specified for sub-clustering analyses. Uniform manifold approximation and projection (UMAP) was used for two-dimensional visualization of transcriptional relationships between cells. Cell populations were annotated manually based on canonical lineage-specific marker genes together with reference to previously published human milk and PBMC single-cell datasets. T cells were identified by expression of *CD3D, CD3E*; B cells by *CD79A, MS4A1*; NK cells by *NKG7*; neutrophils by *CXCL8, S100A8, S100A9*; macrophages/monocytes by *CD68, CD63, MARCO*; endothelial cells by *PECAM1*; platelets by *PPBP* and *PF4*; and lactocytes by *LALBA, CSN2.* Lactocyte subsets were further distinguished based on differential *KLF6* expression patterns.

Sub-clustering analyses were performed for T cells, macrophages/monocytes, and lactocyte populations to define finer functional states at a resolution of 0.3. T-cell subsets were annotated using canonical markers: CD4 T cells – *CD3D, CD3E, CD3G, CD4; CD8* T cells – *CD3D, CD3E, CD3G, CD8A, CD8B;* Central memory T cells – *CCR7, CD69*; Effector memory T cells – *GZMB*; Cytotoxic CD8 T cells – *GZMB, GNLY, PRF1*; γδ T cells – *TRDV1, TRGV9, TRDC*; Regulatory T cells *– FOXP3, IL2RA, CTLA4, TIGIT*; innate-like – *KLRB1, RDC, TRGC1*; Double-negative T cells – *CD3* positive, *CD4/CD8* negative. Monocyte and macrophage subsets were defined as: Classical monocytes – *CD14, LYZ, VCAN, FCGR3A; Macrophage 1 – TREM2, CD9, APOC1, APOE*; Macrophage 2 *– ITGAX, CLEC7A, CD163, CCL7, CXCL1, IL1A* (10, 22, 24). Milk neutrophils were subset from the integrated Seurat object based on canonical marker expression (e.g., *S100A8/A9, CXCL8*) and annotated cellType labels. Differential expression between lactation stages (colostrum vs mature; transition included for visualization) was performed using Seurat FindMarkers (Wilcoxon rank-sum test; min.pct = 0.1; logfc.threshold = 0; FDR correction), and top differentially expressed genes were visualized by heatmap. Differential gene expression analyses were performed using Wilcoxon rank-sum testing with Benjamini–Hochberg false discovery rate correction. Genes with adjusted p-values < 0.05 were considered statistically significant. Only genes expressed in at least 10% of cells within a cluster and with a minimum log fold-change >0.25 were included for downstream interpretation.

In general, functional pathway enrichment analyses were performed using clusterProfiler v3.6.0 based on Gene Ontology biological process databases. Functional module scores for neutrophil antimicrobial programs, chemotaxis, NETosis, T-cell effector function, memory signatures, and regulatory programs were calculated using AddModuleScore in Seurat based on curated gene signatures derived from published immune transcriptional datasets (10, 22, 24). Functional interpretation of stage-associated neutrophil transcriptional changes was further supported by Gene Ontology Biological Process (GO BP) enrichment performed separately on genes upregulated in colostrum and in mature milk neutrophils (FDR-adjusted enrichment), visualized as dot plots showing gene ratio and term counts. To visualize stage-associated remodelling within neutrophils, we generated DotPlots displaying the percent of expressing cells and scaled average expression of representative antimicrobial/innate effector genes versus IFN/regulatory-associated genes across lactation. T cell functional states were quantified using Seurat AddModuleScore function with curated gene sets representing effector and memory programs. Module scores were compared across lactation stages using Wilcoxon rank-sum testing with Benjamini–Hochberg false discovery rate correction.

Exploratory ligand–receptor interaction analysis was performed using CellChat v1.6.1 to infer predicted intercellular communication networks across lactation stages. As these analyses represent computational inference without experimental validation, they were interpreted as hypothesis-generating observations only.

#### 4.4.2 Flow cytometry

In the validation phase, cell pellets of human milk were then washed, resuspended in 0.1-2.0 mL of MACS buffer (Miltenyi) to approximately 1 million cells/mL, for optimal cell to antibody staining ratios and stained with antibody panels for CD45^+^ and lineage markers (**Supplementary Table 5**). The final dilution of antibody used was determined through titration experiments. Samples were fixed with Fixation/Permeabilization Buffer from eBioscience™ staining buffer set and stored at 4 °C protected from light until analysis by flow cytometry, all samples were analysed within 24 hours using high-dimensional flow cytometry (Cytek Aurora).

Samples were acquired on a Cytek Aurora spectral flow cytometer configured with 5 lasers (355 nm, 405 nm, 488 nm, 561 nm, and 640 nm). Before sample analysis, the flow cytometer settings were checked using Cytometer Setup and Tracking beads (CS&T beads, BD) according to the manufacturer’s instructions. Spectral reference controls were generated using single-stained compensation beads for each fluorochrome. Spectral unmixing and autofluorescence extraction were performed in SpectroFlo software prior to downstream analysis in FlowJo software (version 10.1.0, Tree Star, Ashland, OR, USA). The same unmixing matrix was applied across samples acquired within the same experimental batch. Gating strategy is shown in **Supplementary Figure 2a**. The flow cytometry validation panel was designed to assess major immune lineage populations and did not include dedicated phenotypic characterization of macrophage or monocyte subsets. Detection of rare subsets such as γδ T cells and regulatory T cells by flow cytometry was not feasible in most samples due to limited total cell numbers.

#### 4.4.3 Multiplex microbead-based immunoassay (luminex assay) analysis

Human milk supernatant samples were collected for Luminex analysis using the ProcartaPlex™ Customised 22-Plex (ThermoFisher), TGF beta 1 Human ProcartaPlex™ Simplex kit (Cat. No. EPX01a-10249-901) and Lactoferrin Human ProcartaPlex™ Simplex kit (EPX010-122520-901). Both Simplex kits are used together with ProcartaPlex Human Basic Kit (Cat. No. EPX010-10420-901). The 22-plex Luminex kit analyte detection panel includes BLC (CXCL13), IL-1b, IL-2, IL-4, IL-5, IP-10 (CXCL10), IL-6, IL-7, IL-8, IL-10, IFN-b, G-CSF, IFN-g, GM-CSF, TNF-a, I-TAC, IFN-a, MIG (CXCL9), ENA-78, MIP-2a, IL-2/IL-23p40, IL-21. Samples were run neat without dilution.

Prior to measuring TGF-b quantitatively, the samples were activated. 10 uL of 1N HCL was added to 40 uL of sample and mixed well. The mixture was incubated for 10 minutes at room temperature. After that, 8ul of 1.2N NAOH/0.5M HEPES was added to neutralize acidified the sample and mixed well. The final dilution factor will be 1.45x. As for Lactoferrin, samples were diluted with universal assay buffer (provided in kit) 100,000x prior to assay.

Samples and standards were incubated overnight at 4 °C with fluorescent-coded magnetic beads pre-coated with capture antibodies in a 96-well plate. Plates were washed twice and incubated sequentially with biotinylated detection antibodies (1 hour) and streptavidin–PE (30 minutes), with washes between steps. Bead complexes were resuspended in sheath fluid and acquired on an xMAP INTELLIFLEX DR-SE system using INTELLIFLEX software. Data were analysed with Bio-Plex ManagerTM 6.1.1 (Bio-Rad), and standard curves were generated using 4- or 5-parameter logistic regression to derive mean fluorescence intensity and concentration values.

Although 22 analytes were initially measured, only cytokines with concentrations above the lower limit of detection in ≥70% of samples within at least one stage were retained for analysis. This filtering resulted in a final panel of 14 cytokines: CXCL13 (BLC), IL-1β, CXCL10 (IP-10), TNF-α, CXCL8, IL-21, CXCL5, TGF-β, IL-6, CXCL9, IL-12, CXCL2, CXCL11, and IL-7. Cytokines outside this detectable range were excluded from statistical and multivariate analyses.

Raw Luminex cytokine concentration data (pg/mL) were imported into R and annotated with donor ID and lactation stage. Values below the lower limit of detection were replaced with LLOD/√2, and concentrations were log10-transformed to stabilize variance. Data were then z-score–scaled per cytokine to enable comparison across analytes. Stage-wise summaries were generated and visualized as heatmaps using pheatmap. Group comparisons across lactation stages were performed on log-transformed values using Kruskal–Wallis tests with *post hoc* Wilcoxon rank-sum tests and Benjamini–Hochberg correction.

Principal component analysis (PCA) was performed on the scaled cytokine matrix using prcomp() with singular value decomposition. Sample scores for PC1 and PC2 were extracted and annotated with donor and lactation stage metadata. Cytokine contributions were obtained from the PCA rotation matrix. PCA plots were generated in ggplot2, with samples coloured by stage and 95% confidence ellipses added using stat_ellipse(). Cytokine loading vectors were visualized as arrows using ggrepel, with key contributors highlighted. Axes were labelled with the percentage variance explained. To align soluble mediator measurements with cellular sources and targets, we assessed expression of selected cytokine/chemokine ligands (CXCL8, CXCL10, IL7, TGFB1, CXCL13, LTF) and their corresponding receptors (CXCR1/2/3/5, IL7R, IL2RG, TGFBR1/2) across major milk cell populations and lactation stages using stage-resolved DotPlots.

### 4.5 Data visualization and statistics

For statistical comparisons between two groups, we used unpaired t tests or non-parametric equivalents as appropriate. For analyses involving more than two groups, Kruskal–Wallis tests with Dunn *post-hoc* correction was applied as the primary approach.

Because some donors in the validation phase contributed samples at multiple lactation stages, sensitivity analyses accounting for repeated measures were performed using linear mixed-effects models with donor as a random intercept, and, in donors with complete matched samples, Friedman tests with paired Wilcoxon comparisons. These repeated-measures analyses produced results consistent with the primary findings.

When mixed-effects modelling was not feasible, stage-level donor summaries were analysed to avoid pseudo-replication. Unless otherwise specified in figure legends, figures display nonparametric stage-wise comparisons treating samples as independent observations. All tests were two-sided. Sample accounting and participant flow are provided in **Supplementary Table 4** and **Supplementary Figure 1**. Schematic representations of experimental workflow were created using BioRender (https://www.biorender.com).

### 4.6 Study approval

Participants provided written informed consent and completed a demographic questionnaire. The study was approved by the National Healthcare Group Domain Specific Review Board, Singapore (DSRB Ref: 2023/00416).

## Data Availability

The data associated in the study are deposited in the NCBI repository, accession number GSE335791. The raw sequencing data have been deposited in the NCBI Sequence Read Archive (SRA) under BioProject PRJNA1477497, and the processed single-cell transcriptomic data and associated metadata have been deposited in GEO under accession GSE335791.

## Supplementary material

Supplementary Figure 1 CONSORT-style flow diagram of participant recruitment and sample flow. Thirty mothers were enrolled in the MILK cohort. Six donors formed the discovery phase, contributing 10 milk samples for single-cell RNA sequencing, including four paired maternal blood samples. Thirty donors comprised the validation phase, providing 45 milk samples for flow cytometry and cytokine profiling. Validation samples included Colostrum: 9 samples from 7 donors; Transitional: 23 from 12 donors; Mature: 13 from 13 donors. Donors could contribute samples at multiple stages.

Supplementary Figure 2 Flow cytometric profiling of immune cells and cytokines across lactation. **(A)** Representative gating strategy identifying live *CD45* leukocytes, neutrophils (*CD15CD66b*), T cells (*CD3*), B cells (*CD19*), and *CD4* and *CD8* T cell subsets. **(B)** UMAP and proportion of macrophage subsets in human milk across lactation (n=10). **(C)** UMAP and proportion of lactocyte subsets in human milk across lactation (n=10). **(D)** Frequencies of *CD4* and *CD8* T cells across lactation stages (n = 30) (ns across lactation for *CD4, CD8* respectively). **(E)** Frequencies of *CD3 T* cells and *CD19* B cells across lactation stages (n = 30). (ns across lactation for *CD3, CD8* respectively). Boxplots show median and interquartile range. Each dot represents one sample. Statistical comparisons across lactation stages were performed using Kruskal–Wallis tests with Benjamini–Hochberg-adjusted Dunn’s multiple-comparisons test. Only statistically significant comparisons are shown (*p < 0.05).

Supplementary Figure 3 **(A)** Comparison of CD45+ (immune) cell percentages across stages via scRNA-sequencing (colostrum n= 4; transitional n = 2; mature n=4 samples); Neutrophil-to-lymphocyte ratio (NLR) across lactation stages measured by **(B)** scRNA-sequencing (colostrum n= 4; transitional n = 2; mature n=4 samples), and **(C)** flow cytometry (colostrum n= 9; transitional n= 23, mature n=13 samples). Dashed red lines indicate NLR = 1; Boxplots show median and interquartile range. Each dot represents one sample. Statistical comparisons across lactation stages were performed using Kruskal–Wallis tests with Benjamini–Hochberg-adjusted Dunn’s multiple-comparisons test. Only statistically significant comparisons are shown (ns = not significant; *p < 0.05).

Supplementary Figure 4 Gene Ontology (GO) biological processes (BP) for neutrophils in milk. **(A)** GO BP for neutrophils whereby processes are upregulated in colostrum compared to mature milk **(B)** GO BP for neutrophils whereby processes are upregulated in mature milk compared to colostrum.

Supplementary Figure 5 Ligand–receptor landscape across lactation. Dot plots showing expression of selected chemokines, cytokines, and corresponding receptors across major milk cell populations at colostrum, transitional, and mature stages. Dot size represents percentage of cells expressing the gene; colour intensity represents scaled average expression within each gene. Shaded regions denote lactation stage blocks. These data support the stage-dependent shift in immune programming described in the main text.

Supplementary Figure 6 Progressive intensification of lactocyte 2 to T cell communication across lactation stages. **(A)** Communication network for colostrum amongst cells where Lactocyte 2, emerge as dominant signal senders. Node size reflects cell abundance, and edge width represents the strength of predicted ligand–receptor interactions. **(B)** Communication network for transition stage. **(C)** Communication network for mature stage. **(D)** Sender–receiver interaction matrices stratified by lactation stage, illustrating dynamic remodelling of intercellular communication, with increased inferred lactocyte-associated signalling in mature milk. Lactocyte 2 show increasing signalling strength to T cells across lactation.

Supplementary Figure 7 Cell-cell chat, heatmap of outgoing signalling pattern across cell types and Gene ontology enrichment analysis of genes upregulated in colostrum compared with mature milk. **(A)** Overall cell–cell communication network inferred by CellChat, showing interactions between major immune and epithelial populations. Node size reflects cell abundance, and edge width represents the strength of predicted ligand–receptor interactions. **(B)** Communication network for colostrum through MHC-I signalling pathway network **(C)** Communication network for transitional stage through MHC-I signalling pathway network. **(D)** Communication network for mature stage through MHC-I signalling pathway network, whereby T cells showed increasing inferred outgoing signalling strength in mature milk. **(E)** Heatmap of outgoing signalling patterns across cell types, highlighting pathway-specific contributions (including MHC-I, MHC-II, GRN, SPP1, MIF, VISFATIN, ICAM, TNF, TGF-β, CXCL, and CCL). Colour intensity denotes relative signalling strength. **(F)** Gene ontology enrichment analysis of genes upregulated in colostrum compared with mature milk, highlighting biological processes related to immune activation, chemotaxis, metabolism, and cellular localization. Dot size represents gene count, and colour indicates adjusted P value.

Supplementary Table 1 Maternal and neonatal clinical characteristics of the study cohort (n = 30).

Supplementary Table 2 Sample accounting across lactation stages of the discovery phase. Discovery phase (donor-level detail) whereby there are 6 donors, with 10 milk samples and 4 paired maternal blood samples for scRNA-sequencing.

Supplementary Table 3 Sample accounting across lactation stages of the validation phase. Validation phase whereby analyses performed on 45 milk samples (n = 30 donors total) using high-dimensional flow cytometry and Luminex cytokine profiling respectively (includes the 6 discovery phase + 24 validation-only donors.

Supplementary Table 4 scRNA-sequencing quality control metrics by sample.

Supplementary Table 5 Flow cytometry lineage markers.
