# Supplementary material for: Functional shifts in immune composition follow lactation stage in human milk
Source: Front Immunol. 2026 Jun 24;17:1850094. doi: 10.3389/fimmu.2026.1850094 (PMC13343403; doi:10.3389/fimmu.2026.1850094)
Supplement: Supplementary Figure 1 — CONSORT-style flow diagram of participant recruitment and sample flow. Thirty mothers were enrolled in the MILK cohort. Six donors formed the discovery phase, contributing 10 milk samples for single-cell RNA sequencing, including four paired maternal blood samples. Thirty donors comprised the validation phase, providing 45 milk samples for flow cytometry and cytokine profiling. Validation samples included Colostrum: 9 samples from 7 donors; Transitional: 23 from 12 donors; Mature: 13 from 13 donors. Donors could contribute samples at multiple stages. [file DataSheet1.docx]

Supplementary Material

# Supplementary Figures and Tables

## Supplementary Figures


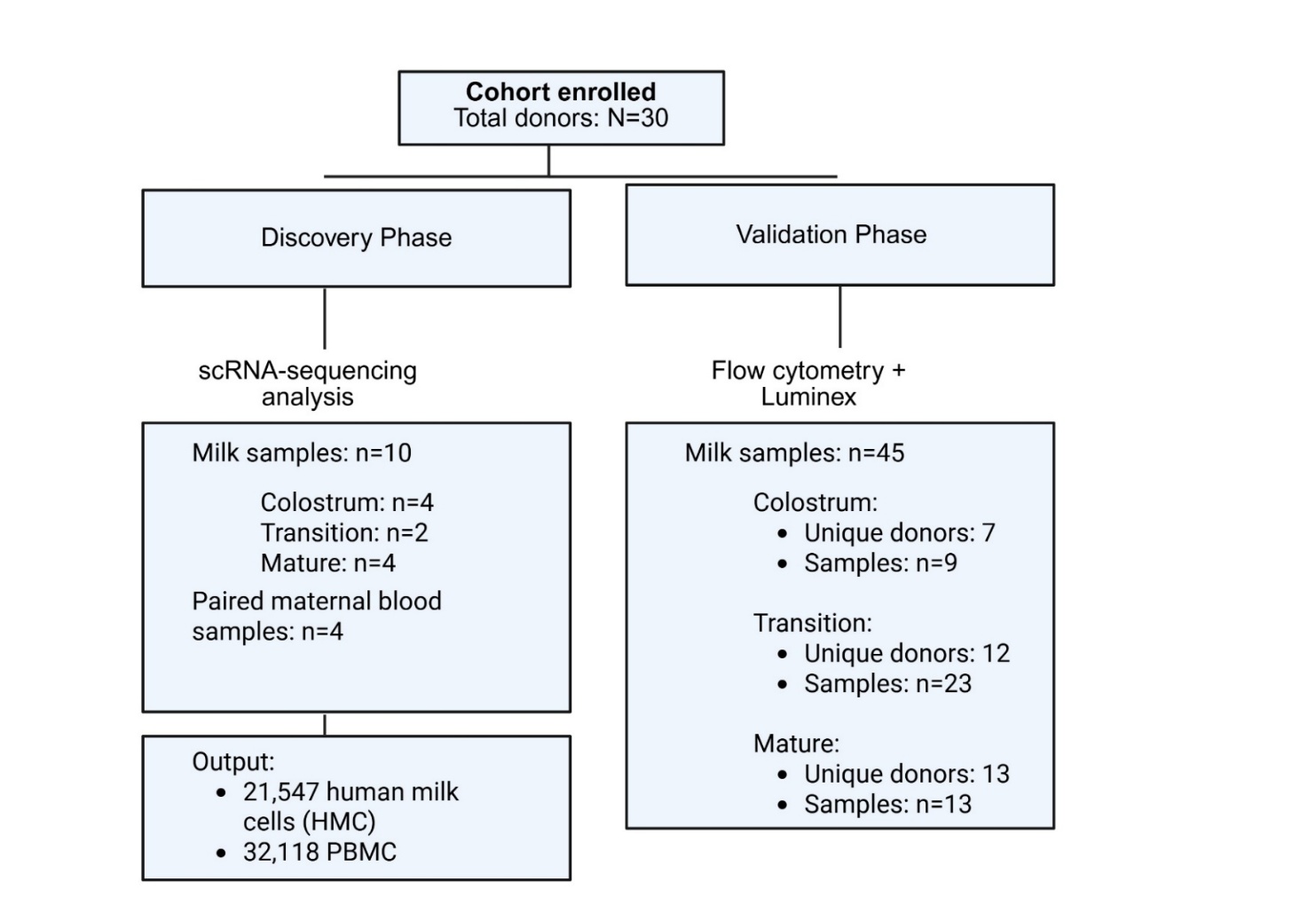


**Supplementary Figure 1.** **CONSORT-style flow diagram of participant recruitment and sample flow.** Thirty mothers were enrolled in the MILK cohort. Six donors formed the discovery phase, contributing 10 milk samples for single-cell RNA sequencing, including four paired maternal blood samples. Thirty donors comprised the validation phase, providing 45 milk samples for flow cytometry and cytokine profiling. Validation samples included Colostrum: 9 samples from 7 donors; Transition: 23 from 12 donors; Mature: 13 from 13 donors. Donors could contribute samples at multiple stages.

**
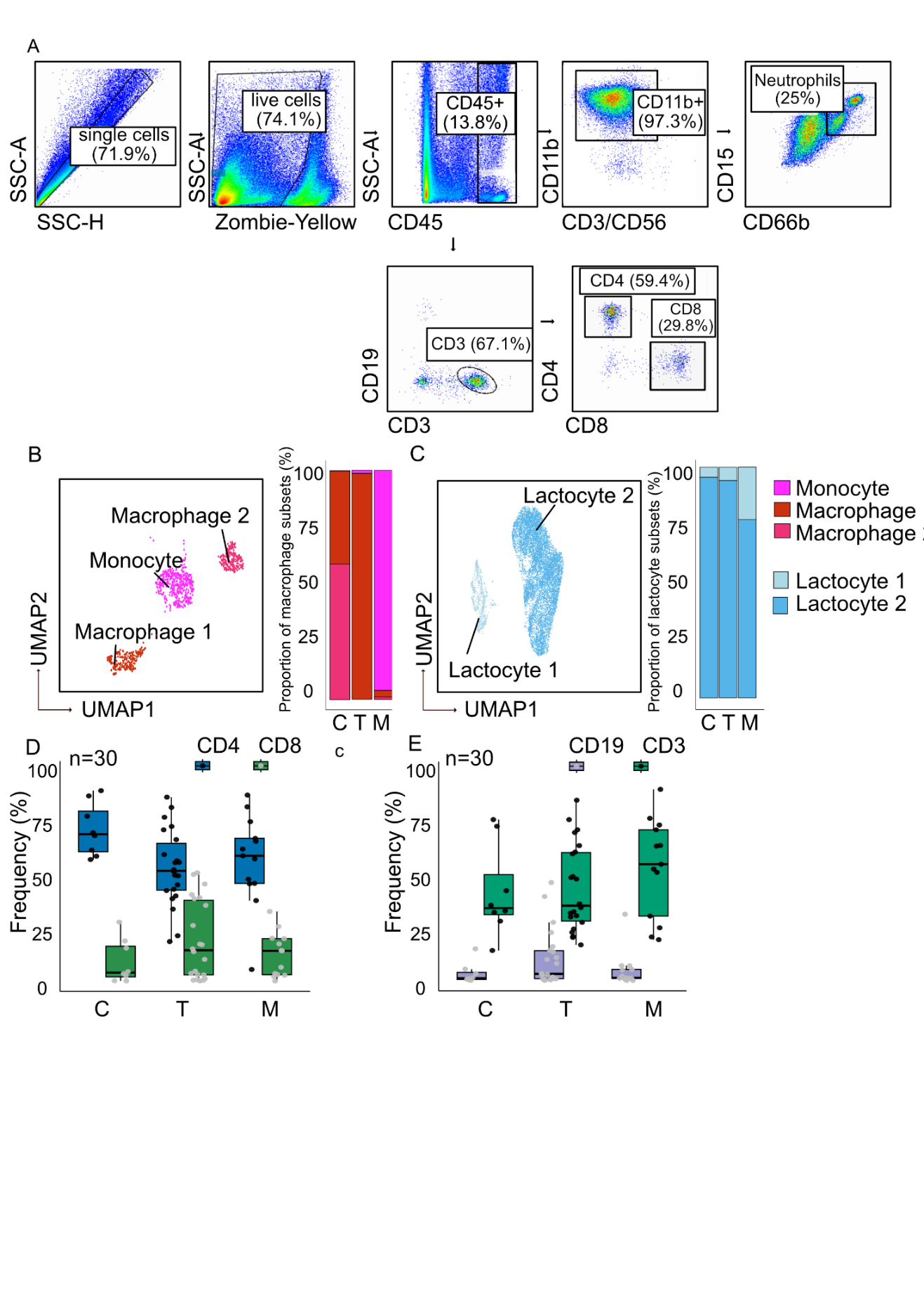
**

**Supplementary Figure 2**. **Flow cytometric profiling of immune cells and cytokines across lactation.** (A) Representative gating strategy identifying live *CD45* leukocytes, neutrophils (*CD15CD66b*), T cells (*CD3*), B cells (*CD19*), and *CD4* and *CD8* T cell subsets (B) UMAP and proportion of macrophage subsets in human milk across lactation (n=10) (C) UMAP and proportion of lactocyte subsets in human milk across lactation (n=10) (D) Frequencies of *CD4* and *CD8* T cells across lactation stages (n = 30) (ns across lactation for *CD4, CD8* respectively) (E) Frequencies of *CD3 T* cells and *CD19* B cells across lactation stages (n = 30). (ns across lactation for *CD3, CD8* respectively). Boxplots show median and interquartile range. Each dot represents one sample. Statistical comparisons across lactation stages were performed using Kruskal–Wallis tests with Benjamini–Hochberg-adjusted Dunn’s multiple-comparisons test. Only statistically significant comparisons are shown (*p < 0.05)


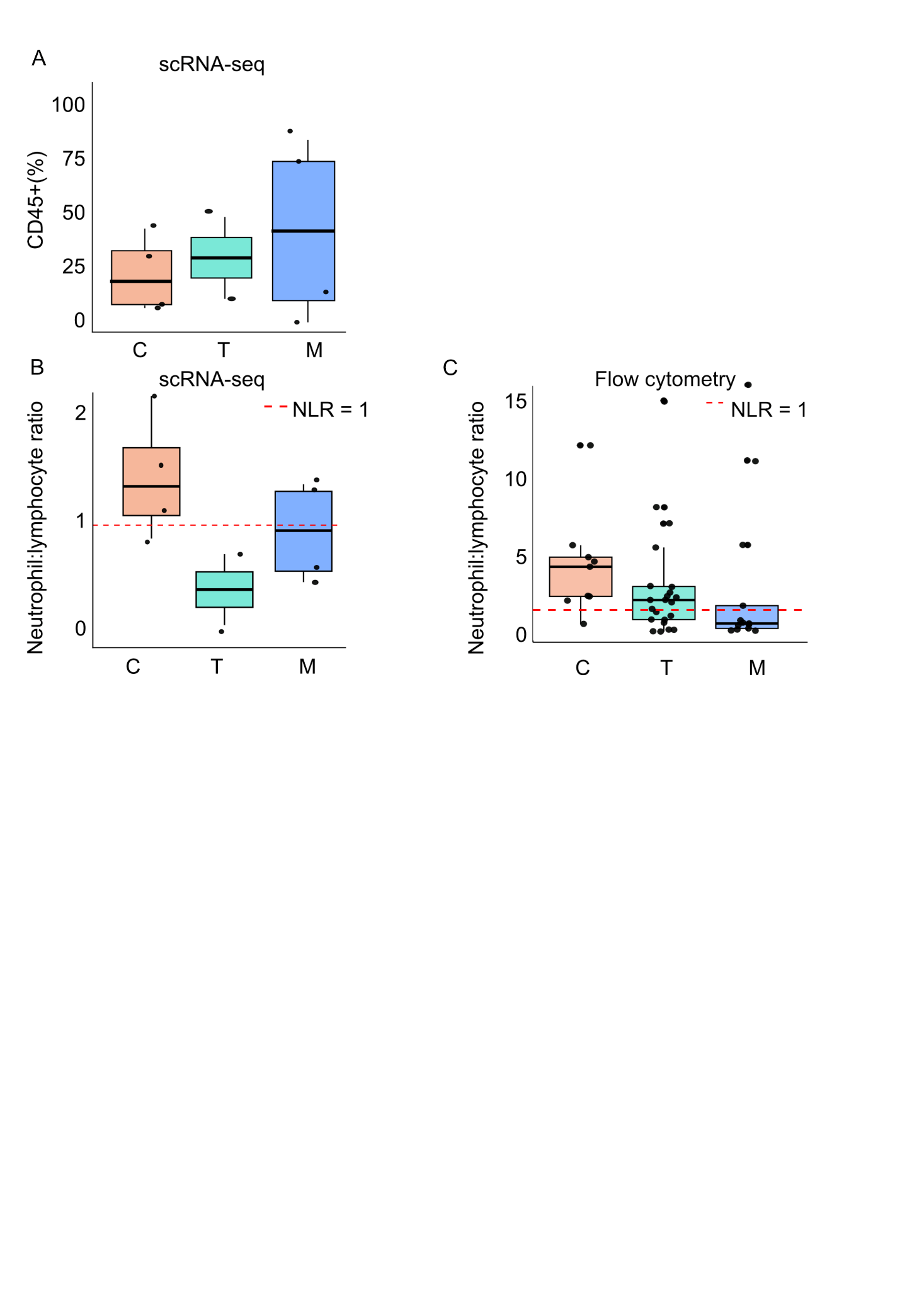


**Supplementary Figure 3**. (A) Comparison of CD45+ (immune) cell percentages across stages via scRNA-sequencing (colostrum n= 4; transitional n = 2; mature n=4 samples); Neutrophil-to-lymphocyte ratio (NLR) across lactation stages measured by (B) scRNA-sequencing (colostrum n= 4; transitional n = 2; mature n=4 samples), and (C) flow cytometry (colostrum n= 9; transitional n= 23, mature n=13 samples). Dashed red lines indicate NLR =1; Boxplots show median and interquartile range. Each dot represents one sample. Statistical comparisons across lactation stages were performed using Kruskal–Wallis tests with Benjamini–Hochberg-adjusted Dunn’s multiple-comparisons test. Only statistically significant comparisons are shown (ns = not significant; *p < 0.05)

**
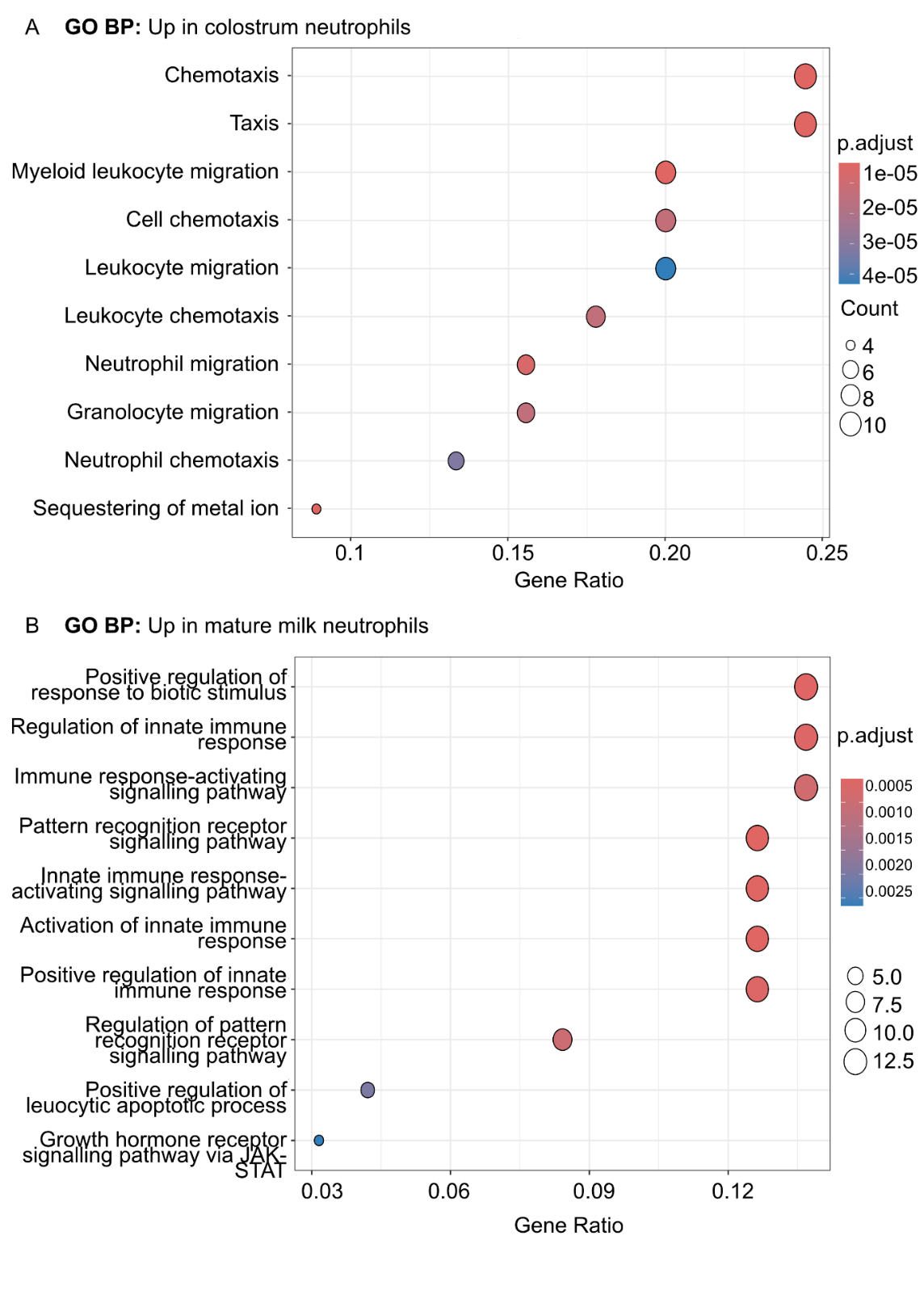
**

**Supplementary Figure 4. Gene Ontology (GO) biological processes (BP) for neutrophils in milk. (A)** GO BP for neutrophils whereby processes are upregulated in colostrum compared to mature milk **(B)** GO BP for neutrophils whereby processes are upregulated in mature milk compared to colostrum

**
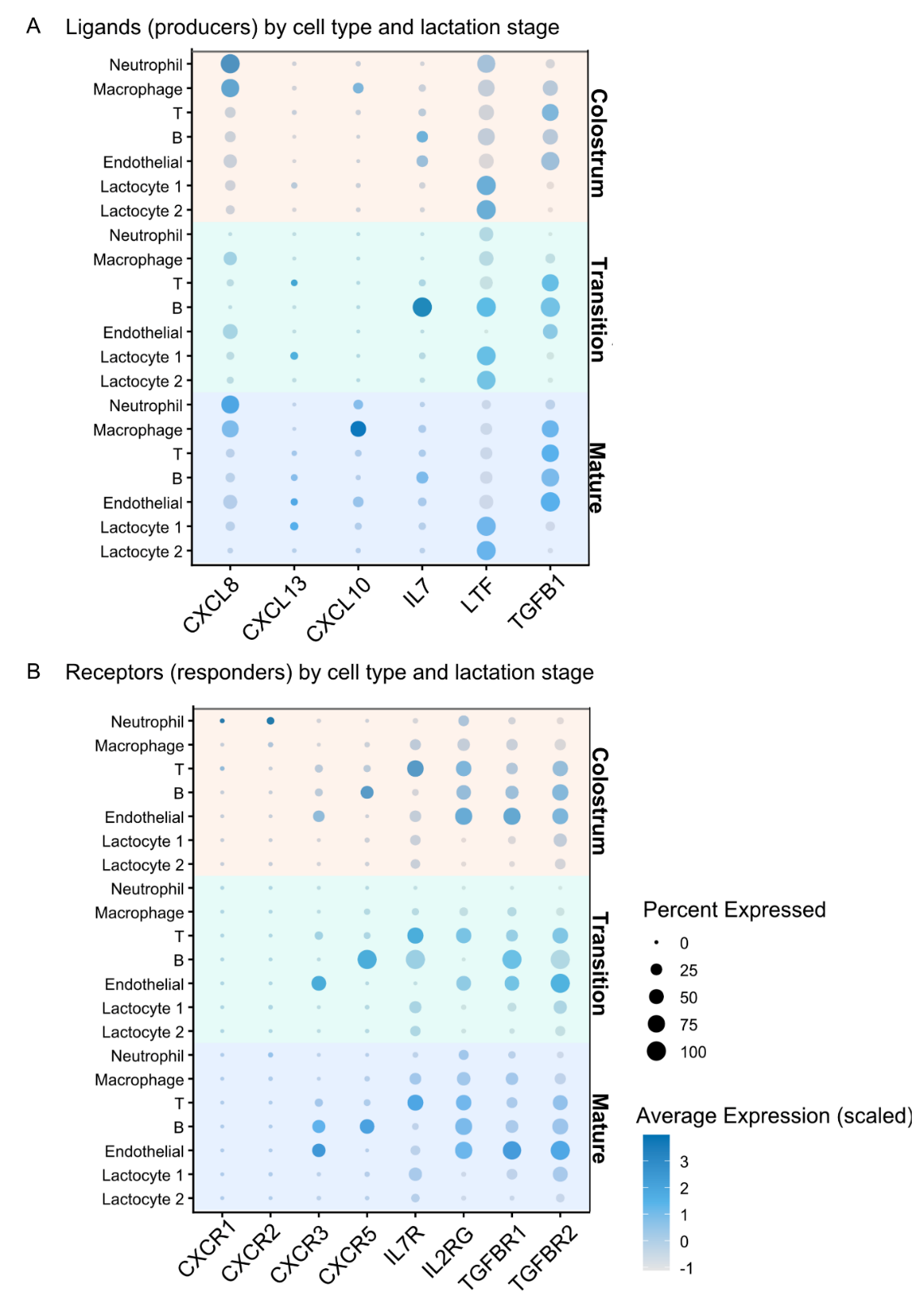
**
**Supplementary Figure 5. Ligand–receptor landscape across lactation.** Dot plots showing expression of selected chemokines, cytokines, and corresponding receptors across major milk cell populations at colostrum, transition, and mature stages. Dot size represents percentage of cells expressing the gene; colour intensity represents scaled average expression within each gene. Shaded regions denote lactation stage blocks. These data support the stage-dependent shift in immune programming described in the main text.

**
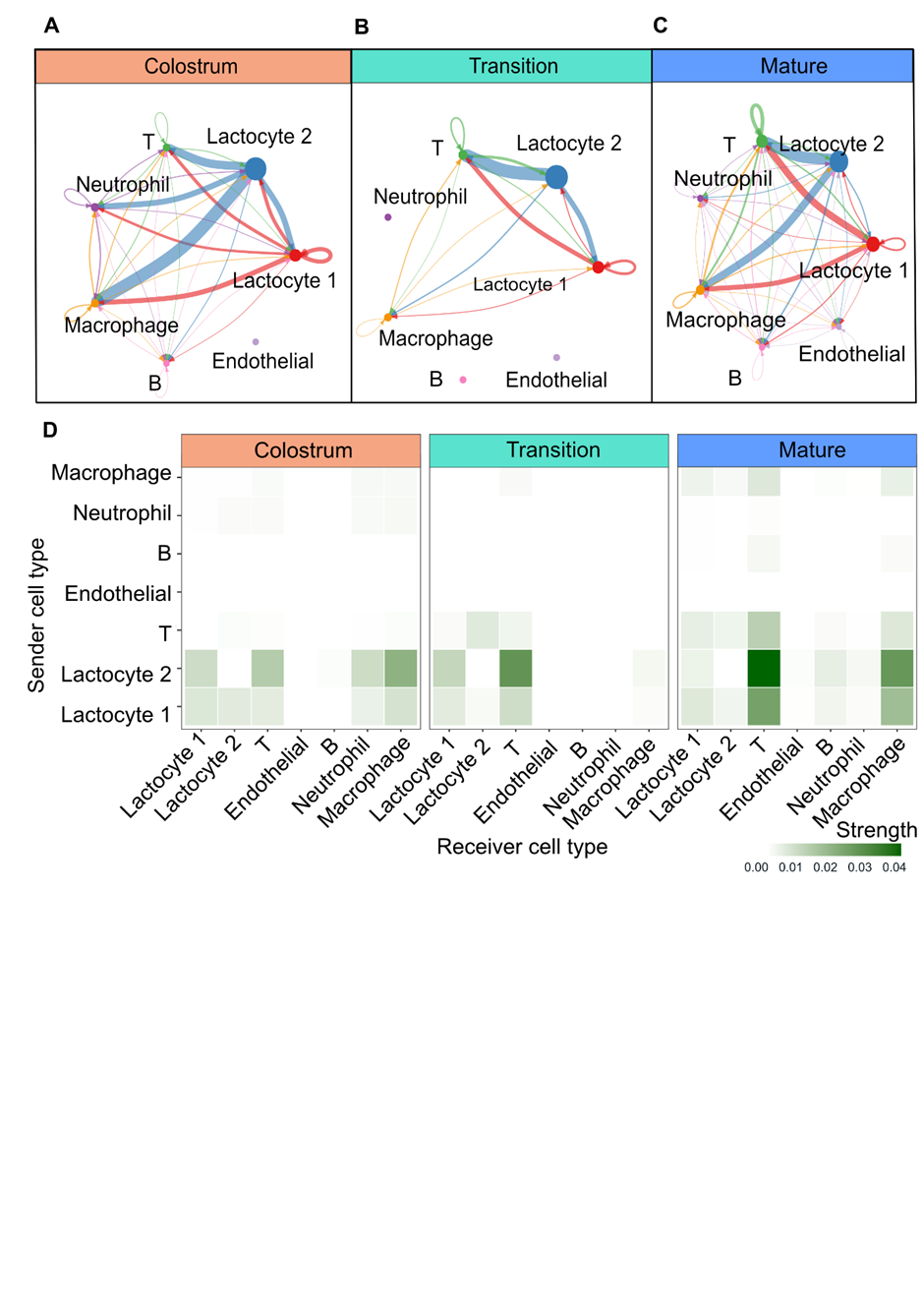
**

**Supplementary Figure 6. Progressive intensification of lactocyte 2 to T cell communication across lactation stages**

**(A)** Communication network for colostrum amongst cells where Lactocyte 2, emerge as dominant signal senders. Node size reflects cell abundance, and edge width represents the strength of predicted ligand–receptor interactions.

**(B)** Communication network for transition stage

**(C)** Communication network for mature stage

**(D)** Sender–receiver interaction matrices stratified by lactation stage, illustrating dynamic remodelling of intercellular communication, with increased inferred lactocyte-associated signalling in mature milk. Lactocyte 2 show increasing signalling strength to T cells across lactation

**
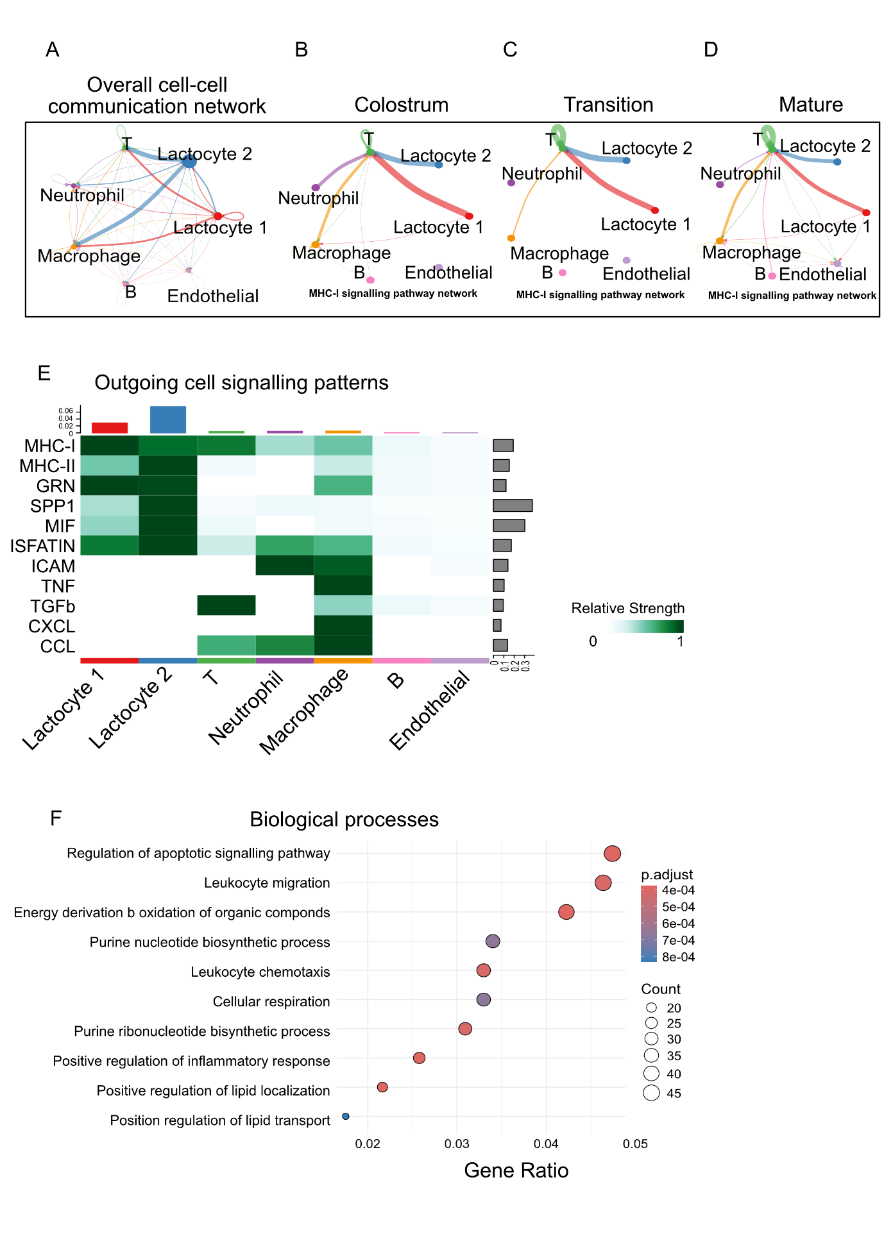
**

**Supplementary Figure 7.** Cell-cell chat, heatmap of outgoing signalling pattern across cell types and Gene ontology enrichment analysis of genes upregulated in colostrum compared with mature milk. **(A)** Overall cell–cell communication network inferred by CellChat, showing interactions between major immune and epithelial populations. Node size reflects cell abundance, and edge width represents the strength of predicted ligand–receptor interactions. **(B)** Communication network for colostrum through MHC-I signalling pathway network **(C)** Communication network for transition stage through MHC-I signalling pathway network **(D)** Communication network for mature stage through MHC-I signalling pathway network, whereby T cells showed increasing inferred outgoing signalling strength in mature milk **(E)** Heatmap of outgoing signalling patterns across cell types, highlighting pathway-specific contributions (including MHC-I, MHC-II, GRN, SPP1, MIF, VISFATIN, ICAM, TNF, TGF-β, CXCL, and CCL). Colour intensity denotes relative signalling strength. **(F)** Gene ontology enrichment analysis of genes upregulated in colostrum compared with mature milk, highlighting biological processes related to immune activation, chemotaxis, metabolism, and cellular localization. Dot size represents gene count, and colour indicates adjusted P value.

## Supplementary Tables

**Supplementary Table 1. Maternal and neonatal clinical characteristics of the study cohort (n=30)**

| **Clinical Characteristics** | **n = 30** |
| --- | --- |
| Maternal age, years (mean, 95% confidence interval (CI)) | 33.3 (32.7 – 34.0) |
| Maternal Body Mass Index (BMI) (mean, 95% CI) | 28.1 (24.5 – 28.1) |
| Maternal past medical history, if any, n (%) |  |
| Maternal gestational diabetes | 5 (16.7) |
| Maternal pre-eclampsia/pre-existing hypertension | 0 (0) |
| Maternal infections/bacterial colonization, if any, n (%) |  |
| Group B streptococcus (GBS) high vaginal swab positive | 7 (23.3) |
| Methicillin-resistant Staphylococcus Aureus (MRSA) carriage | 1 (3.3) |
| Histologically proven maternal chorioamnionitis | 2 (6.6) |
| Antenatal vaccinations, if any, n (%) |  |
| Influenza vaccine | 22 (73.3) |
| Tetanus/Diphtheria/Pertussis vaccine | 26 (86.7) |
| COVID-19 vaccination | 1 (3.3) |
| First child, n (%) | 8 (26.7) |
| Gender of child, female: male ratio | 15:15 |
| Mode of delivery, n (%) |  |
| Normal vaginal delivery | 18 (60) |
| Lower segment Caesarean delivery | 12 (40) |
| Gestational age at birth, weeks, mean (95% CI) | 38+3 (37+2 – 39+3) |
| Birth weight of child, g, mean (95% CI) | 2985 (2762 – 3208) |
| Length of child at birth, cm, mean (95% CI) | 47.1 (43.6 – 50.6) |
| Occipital-frontal circumference of child at birth, cm, mean (95% CI) | 33.4 (32.4 – 34.4) |

**Supplementary Table 2. Sample accounting across lactation stages of the discovery phase**

Discovery phase (donor-level detail) whereby there are 6 donors, with 10 milk samples and 4 paired maternal blood samples for scRNA-sequencing

| **Donor ID** | **Phase** | **Colostrum sample (D0–6)** | **Transition sample (D7–20)** | **Mature sample (D21–60)** | **Paired maternal blood sample** | **Included in scRNA-sequencing** | **Included in Flow Cytometry as part of Validation phase** | **Included in Luminex as part of Validation phase** |
| --- | --- | --- | --- | --- | --- | --- | --- | --- |
| D1 | Discovery | × |  | × | × | × | × | × |
| D2 | Discovery | × |  | × | × | × | × | × |
| D3 | Discovery | × |  | × | × | × | × | × |
| D4 | Discovery | × |  | × | × | × | × | × |
| D5 | Discovery |  | × |  | – | × | × | × |
| D6 | Discovery |  | × |  | – | × | × | × |

**Supplementary Table 3. Sample accounting across lactation stages of the validation phase**

Validation phase whereby analyses performed on 45 milk samples (n = 30 donors total) using high-dimensional flow cytometry and Luminex cytokine profiling respectively (includes the 6 discovery phase + 24 validation-only donors

| **Stage** | **Unique donors (per stage)** | **No. of samples** |
| --- | --- | --- |
| Colostrum | 7 | 9 |
| Transition | 12 | 23 |
| Mature | 13 | 13 |
| Total | 30 donors | 45 samples |
| **Note:** Unique donor counts are stage-specific; the same donor may contribute samples at multiple stages. Therefore, the sum of donors across stages exceeds the total number of individual donors. | | |

**Supplementary Table 4. scRNA-sequencing quality control metrics by sample**

| **Patient** | **Sample type** | **Lactation stage** | **Cells after QC** | **Median genes/cell** | **Median UMIs/cell** | **Median % mitochondrial** | **Mean genes/cell** | **Mean UMIs/cell** | **Mean % mitochondrial** |
| --- | --- | --- | --- | --- | --- | --- | --- | --- | --- |
| A | Human milk | Colostrum | 4535 | 779 | 1715 | 0.01 | 1220 | 4794 | 0.77 |
| A | Human milk | Mature | 616 | 5559 | 32031 | 4.64 | 4761 | 30689 | 5.20 |
| B | Human milk | Colostrum | 2945 | 734 | 2171 | 0.42 | 1157 | 5133 | 1.51 |
| B | Human milk | Mature | 1568 | 2445 | 7105 | 2.95 | 2630 | 10481 | 3.26 |
| C | Human milk | Colostrum | 1764 | 1770 | 6659 | 0.43 | 2613 | 12688 | 2.24 |
| C | Human milk | Mature | 1360 | 2407 | 8167 | 4.03 | 2859 | 13792 | 4.95 |
| D | Human milk | Colostrum | 3321 | 1825 | 9110 | 0.20 | 1941 | 10406 | 0.55 |
| D | Human milk | Mature | 3217 | 952 | 3485 | 0.00 | 1166 | 5326 | 0.56 |
| E | Human milk | Transition | 238 | 1826 | 6507 | 1.12 | 1969 | 6824 | 2.22 |
| F | Human milk | Transition | 1983 | 1332 | 5691 | 0.09 | 1521 | 7310 | 0.84 |
| A | Blood | Colostrum | 9516 | 2373 | 6726 | 4.47 | 2441 | 7219 | 4.81 |
| B | Blood | Colostrum | 7542 | 2537 | 7491 | 4.32 | 2596 | 7916 | 4.60 |
| C | Blood | Colostrum | 6950 | 2669 | 7722 | 4.26 | 2822 | 8856 | 4.48 |
| D | Blood | Colostrum | 8110 | 2733 | 7189 | 3.23 | 2785 | 7903 | 3.46 |

**Supplementary Table 5. Flow cytometry lineage markers.**

| **Marker** | **Fluorophore** | **Catalogue Number** | **Antibody Clone** | **Company** |
| --- | --- | --- | --- | --- |
| CD3 | BUV395 | 564001 | SK7 | BD Horizon |
| CD4 | BUV496 | 612936 | SK3 | BD Horizon |
| CD8 | FITC | 347903 | 11F2 | BD Biosciences |
| CD45 | APC-Cy7 | 557833 | 2D1 | BD Biosciences |
| CD19 | PE-Cy5 | 555414 | HIB19 | BD Biosciences |
| CD11c | RB780 | 756249 | 3.9 | BD Biosciences |
| Zombie Yellow | BV570 | 423104 | - | Biolegend |
| CD66b | BV421 | 562940 | G10F5 | BD Horizon |
| CD11b | BUV395 | 565976 | M1/70 | BD Biosciences |
